# Engineering Gel-Based Precursors into Advanced ORR Catalysts for Zn–Air Batteries and Fuel Cells: Insights into Hydrogels, Aerogels, Xerogels, Metal–Organic Gels, and Metal Aerogels

**DOI:** 10.3390/gels11070479

**Published:** 2025-06-21

**Authors:** Shaik Gouse Peera, Myunghwan Byun

**Affiliations:** 1 Natural Science Research Institute, College of Natural Sciences, Keimyung University, 1095 Dalgubeol-daero, Daegu 42601, Republic of Korea; 2 Department of Advanced Materials Engineering, College of Engineering, Keimyung University, 1095 Dalgubeol-daero, Daegu 42601, Republic of Korea; myunghbyun@kmu.ac.kr

**Keywords:** gels, oxygen reduction reaction, hydrogels, aerogels, xerogels, metal–organic gels and metal aerogels

## Abstract

Efficient electrocatalysts for the oxygen reduction reaction (ORR) are essential for numerous energy storage and conversion systems, including zinc–air batteries and fuel cells. Cutting-edge Pt/C catalysts remain the most efficient ORR catalysts to date; however, their high cost and inadequate stability impede their use in commercial devices. Recently, transition metal-based electrocatalysts are being pursued as ideal alternatives for cost-effective and efficient materials with a promising future. This review provides an in-depth analysis of the principles, synthesis, and electrocatalytic assessment of noble metal and transition metal-based catalysts derived from diverse gel precursors, including hydrogels, aerogels, xerogels, metal–organic gels, and metal aerogels. Electrocatalysts derived from gel precursors have garnered significant interest due to their superior physicochemical properties, including an exceptionally high surface area, adjustable porosity, adaptability, and scalability. Catalysts obtained from gel precursors offer numerous advantages over conventional catalyst synthesis methods, including the complete utilization of precursors, precise control over surface area and porosity, and uniform distribution of ORR active sites. Among the various types, metal aerogels are distinguished as the superior catalysts, exceeding the Department of Energy’s (DoE) 2025 targets for the mass and specific activities of ORR catalysts. In contrast, hydrogel- and aerogel-derived catalysts excel in terms of ORR activity, specific surface area, and the potential to incorporate high loadings of single-atom catalysts composed of transition metals. Ultimately, we unequivocally categorized the electrocatalysts into high-, moderate-, and low-performance tiers, identifying the most promising catalyst candidate within each gel classification. Concluding insights, future outlooks, and recommendations were provided for the advancement of cost-effective, scalable electrocatalysts derived from gels for fuel cells and zinc–air batteries.

## 1. Introduction

Increasing global warming around the world due to emissions of high levels of carbon dioxide from fossil fuel-driven energy technologies for power generation necessitates the development of alternative energy technologies [1,2]. Several cutting-edge technologies have been investigated, such as lithium-ion batteries, sodium-ion batteries, redox flow batteries, supercapacitors, and solid-state batteries, and sustainable energy harvesting technologies such as microbial fuel cells have been gaining significant interest due to their potential for meeting the demands of next-generation energy storage and conversion systems [3,4,5,6,7,8,9,10,11,12]. In this regard, fuel cells and Zn–air batteries have attracted significant attention in recent years as alternative energy conversion technologies due to their high efficiency and high energy density, respectively [13,14]. Both these technologies work on the principle of electrochemical reactions to generate useful energy. In fuel cells, polymer electrolyte membrane fuel cells, alkaline electrolyte fuel cells, and high-temperature polymer electrolyte membrane fuel cells attract special attention for transportation and stationary applications, whereas Zn–air batteries found their application, such as in electric vehicles and portable electronic devices [15,16]. The efficiency of these technologies primarily depends on the kinetics of the electrochemical reactions that occur on anode and cathode electrodes. While anodic reactions in fuel cells include hydrogen oxidation reactions, in Zn–air batteries, they mainly comprise the oxidation/dissolution of the Zn anode. On the cathodes, both these technologies share a common electrochemical reaction, i.e., the oxygen reduction reaction (ORR). The kinetics of a cathodic ORR, a slow and multistep reaction that necessitates a high kinetic overpotential to overcome the energy barrier, determine the efficiency and power output of fuel cells and Zn–air batteries [17]. The most popular electrocatalyst for ORRs has historically been Pt nanoparticles, supported on high-surface-area carbon (Pt/C) due to its remarkable electrocatalytic activity in reducing oxygen to H_2_O/OH^−^ ions [18]. The efficient ORR electrocatalysts should possess certain characteristics such as (i) an ability to reduce the O_2_/OH^−^ via a dominant direct four-electron reduction, (ii) lower activation energy, especially for a rate-determining step (RDS) and lower Tafel slopes, (iii) an ability to deliver high power density and high electrochemical stability in harsh acidic/alkaline electrolytes, (iv) a high mass and specific activity, (v) stability against carbon corrosion and metal delamination and resistance/mitigation against Fenton reactions, (vi) in case of rechargeable Zn–air batteries/regenerative fuel cells, the electrocatalyst should possess excellent bifunctional activity and lower voltage gaps between charge/discharge reactions, (vii) high density of the ORR active sites that are either nanoparticles of Pt/Pt alloys or single-atom transition metal catalysts that effect the turnover frequency of the ORR, and (viii) in case of alkaline fuel cells/alkaline Zn–air batteries, the electrocatalysts should have resistance to carbonate poisoning. In Zn–air batteries and commercial fuel cells, Pt and Pt alloy catalysts continue to be the best cutting-edge electrocatalysts [19,20]. However, the primary reasons for the significantly restricted commercial applications of fuel cells and reasonably priced Zn–air batteries are the high cost and limited supply of Pt. Thus, it is crucial to look for substitute electrocatalysts that are both kinetically active for ORR and less expensive. Recent research in this area has shown that N-doped carbons (transition metal-N_x_-C) and electrocatalysts composed of transition metals (Fe, Co, Mn, and Ni) are among the most promising catalysts for catalyzing ORR [21,22]. Additionally, a number of studies indicated that M-N_x_-C-based catalysts could be the most effective substitutes for conventional Pt-based catalysts that might be used in commercial applications soon [23].

The stability of the ORR electrocatalysts is essential for sustaining continuous electricity generation over extended durations. It is well known that Pt/C-based catalysts are prone to degradation under electrochemical conditions, leading to the deterioration of ORR activity over time [24,25]. The loss of the electrochemically active surface area (ECSA) is found to be the major cause of loss in ORR activity, which is triggered by a number of phenomena, including carbon corrosion, Pt nanoparticle dissolution/re-deposition, nanoparticle agglomeration, coalescence, and the detachment of the supported nanoparticles [26]. The poor graphitic nature, high density of surface defects, and hydrophilicity of the carbon support due to the presence of O-containing functionalities are some of the drawbacks of Vulcan carbon supports that are used to host Pt nanoparticles [27]. Therefore, researchers proposed several high-corrosion-resistance carbon support alternatives to Vulcan carbon such as graphene, graphitized carbons, carbon nanotubes, multiwalled carbon nanotubes, and several non-carbon-based supports [28,29]. Though the alternative carbon supports present excellent stability against carbon corrosion and related phenomena, they often require prior functionalization either via acid oxidation or non-covalent polymer functionalization to host the Pt nanoparticles [30]. This is due to poor surface defects on the highly graphitized carbon supports, which are essential to evenly disperse the nanoparticles; otherwise, this can lead to agglomerated nanoparticles [31]. On the other hand, non-carbon supports, such as TiO_2_ and SnO_2_, etc., lack essential surface area and have poor electronic conductivity [32]. Therefore, the overall catalyst activity and stability optimization require carefully chosen raw materials, surface functionalization methods, nanoparticle deposition methods, and processing steps to obtain the best alternative ORR catalyst for fuel cells and Zn–air batteries. On the other hand, high active site accessibility, micro/nanoarchitectures, and high conductivity are also indispensable factors for electrocatalyst design, in addition to the catalyst activity and stability. It is well known that the traditional Vulcan carbon possesses micropores, in which deposited nanoparticles are inaccessible to the reactants and cannot participate in the “three-phase boundary” [33]. For the effective mass transfer of reactants to the catalyst surface, the porosity of the carbon support is a major factor. In this context, highly porous and robust multi-dimensional interconnected networks are essential.

Gels, which are non-fluid colloidal substances or polymer networks infused with liquid, exhibit solid-like behavior due to a three-dimensional cross-linked network within the liquid, and have recently garnered significant interest in the field of catalysis [34]. As a result of their porous framework, which contains a large number of defects for mass transfer, high compositional tunability, and ease of synthesis and functionalization, nanostructured gels have emerged as a novel material platform that can be utilized for a variety of applications in the field of energy storage [35]. Electrocatalysts that are derived from gels have exceptional properties such as high specific surface areas and a hierarchical porous structure. These characteristics make it possible for gel networks to potentially host various types of metallic active sites within their porous architectures, which in turn makes the mass transport of the gaseous reactants during electrocatalysis feasible, leading to enhanced electrocatalytic activity [36]. Several types of gels and their derivatives have been explored as excellent electrocatalyst materials, such as (i) hydrogels, (ii) aerogels, (iii) xerogels, (iv) metal–organic gels, and (v) metal gels. Each of these types of gels have their own unique advantages that benefit the electrocatalysis of ORR (Table 1). This review gives a comprehensive overview of five types of gel-derived catalysts for ORR activity in fuel cells and Zn–air batteries, in terms of the synthesis and processing of gels into electrocatalysts, factors that determine the ORR activity of gel-derived Pt and transition metal-based catalysts, the stability of the catalyst in comparison with the traditional Pt/C catalysts, and structure–activity relationship between the gel-derived catalyst and ORR kinetics. A statistical analysis of the gel-derived catalysts was conducted in relation to the commercial standards established by the Department of Energy, highlighting the performance of these catalysts.

## 2. Gel-Derived ORR Catalysts vs. Conventional ORR Catalysts

**Figure 1:**
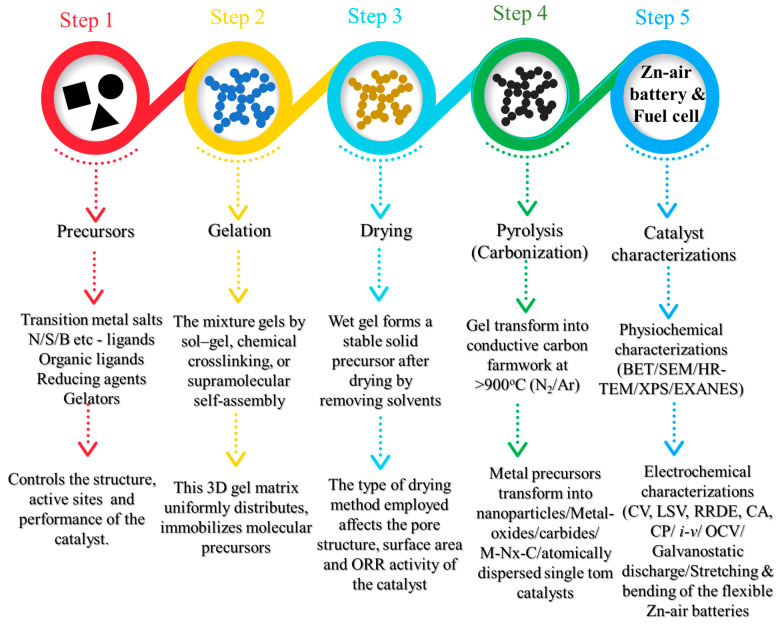
Steps involved in gel–electrocatalyst synthesis.

**Figure 2:**
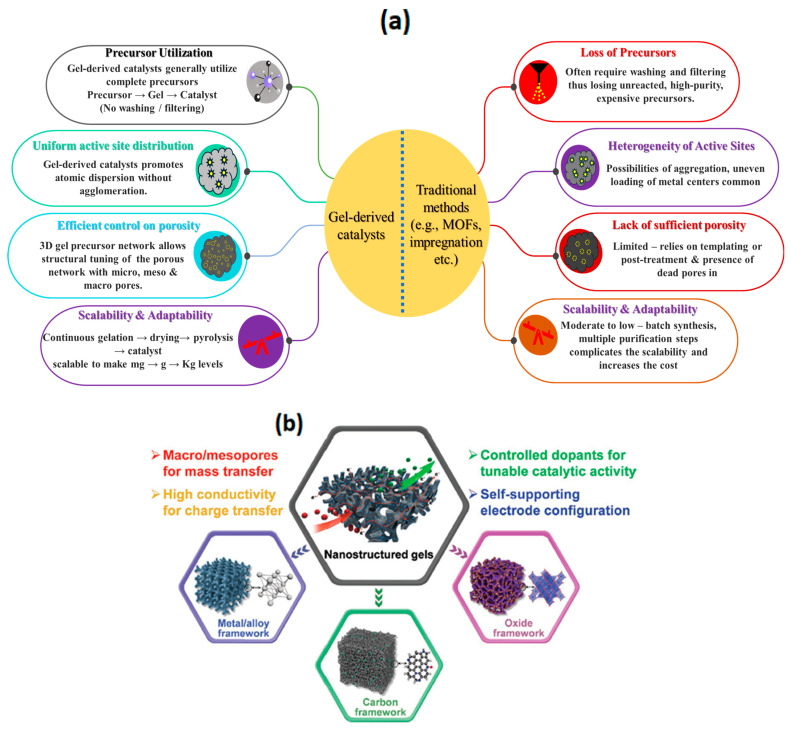
(**a**) Comparison of gel-derived electrocatalysts vs. conventional electrocatalysts. (**b**) Schematic illustration of the key features of gel electrocatalysts [Reproduced with permission from Ref. [36]].

Gel-derived catalysts for ORRs have been gaining continuous interest in recent years due to the unique properties inherited by the catalysts from their precursor gels. Gel-derived catalysts are prepared via the gelation of the precursors, followed by drying the gel. The dried gels are then pyrolyzed to obtain the electrocatalysts (Figure 1). The electrocatalysts that are obtained after pyrolysis of the precursor gels are found to possess excellent three-dimensional networks of porosity, high surface area, high-graphitic-carbon domains, tunable porosity, and the homogeneous distribution of active sites. The gel-derived catalysts offer numerous advantages over traditional catalyst synthesis methods in various aspects (Figure 2a).

### 2.1. Complete Utilization of the Precursors

The gel-derived catalysts generally utilize the complete precursors. Upon gel formation, all precursors are integrated into the gel, which subsequently transforms into the catalyst through drying and pyrolysis. In contrast to traditional synthesis methods like metal–organic framework (MOF)-derived catalysts, solution-based synthesis techniques such as impregnation and surfactant-mediated processes, etc., often necessitate washing and filtering, resulting in the loss of unreacted, high-purity, expensive precursors. Most gel-derived catalyst systems, however, do not require additional washing or filtering, ensuring that all precursors utilized in the synthesis process are retained within the gel matrix. Therefore, it is expected that the cost of gel-derived catalysts could be lower than that of conventional catalysts.

### 2.2. Scalability

Gel-derived catalysts are synthesized via the gelation of the precursors, converting from their solution phase into the solid phase via sol–gel coordination or the self-assembly of precursors. The resulting gels are directly transformed into electrocatalysts via drying and pyrolysis. This entire synthesis process can be simplified and is scalable for producing mg to g to Kg levels, just by allowing continuous gel formation, drying, and pyrolysis, whereas conventional synthesis processes often require multiple steps in the synthesis process, such as filtering, washing, and drying, and sometimes they require special setups such as hydrothermal setups, which complicates the scale-up process.

### 2.3. Uniform Distribution of Active Sites

Unlike traditional synthesis processes such as impregnation, in which it is common to observe the agglomeration of nanoparticles and uneven distribution of active sites, gel-derived catalysts could prevent agglomeration, resulting in the creation of single-atom catalysts with highly dispersed atomic states for enhanced metal active site utilization.

### 2.4. Efficient Control on Surface Area and Porosity

Gel-derived catalysts were found to have excellent 3D porous networks in the catalysts inherited from their 3D network of gel precursors, allowing for the structural tuning of the porous network with a mixture of micro-, meso-, and macropores, whereas in the conventional synthesis process, they require structural directing agents such as surfactants and hard templates, such as mesoporous silica, which requires further chemical treatment to remove the hard templates, complicating the entire process.

### 2.5. Sustainability

In general, gel-derived catalysts are directly transferred to catalysts via drying and pyrolysis; the use of solvents is very minimal, whereas in conventional synthesis, for instance, the synthesis of MOF, the filtering and washing of the products with organic solvents such as methanol are often required, generating large effluents of washing solvents, which require further treatment.

### 2.6. Adaptability

Gel synthesis processes are internal and can be easily tuned by adding additional processors in addition to the precursors of gels, for example, the addition of transition metal salts to the solution of hydrogel, which incorporates and traps the precursor inside the 3D hydrogel network.

## 3. Gel-Derived Catalysts

Gel-derived ORR catalysts have been gaining considerable interest in recent years due to their various advantages over traditional catalysts, as discussed above. The gels are classified based on synthetic route, composition, and the drying of the gels into various distinctive types, each of which possesses unique physicochemical properties. The obtained dried gels can then transfer into electrocatalysts composed of a carbon matrix with heteroatoms such as N, P, B, and S, etc., and the metallic active sites in various phases such as metal oxides, metal sulfides, metal phosphides, metal carbides, and atomically dispersed metallic active sites that serve as ORR active sites (Figure 2b). Broadly, gels are classified into (i) hydrogels, (ii) aerogels, (iii) xerogels, (iv) metal–organic gels (MOGs), and (v) metal gels. The subsequent sections examine the catalysts obtained from each of these gels for the oxygen reduction reaction (ORR). A comprehensive discourse proceeds in a systematic manner, addressing the synthesis processes of the gels, morphological analysis of the catalysts derived from the gels, and their correlation with the kinetics of the oxygen reduction reaction (ORR) and the performance of zinc–air batteries and fuel cells.

### 3.1. Hydrogels

Hydrogels are 3D cross-linked polymer network chains characterized by their ability to hold a large amount of water and swell without dissolving. Hydrogels possess a variety of special characteristics such as mechanical and thermodynamic stability and biocompatibility, due to which hydrogels have found significant applications in the field of drug delivery, sensors, tissue engineering, water purification, batteries, and supercapacitors [37,38,39,40,41,42]. Hydrogels offer 3D hierarchical porous structures with a large accessible surface area that can serve as active sites to host high-density and evenly dispersed nanoparticles and atomically dispersed metallic active sites for the ORR.

#### 3.1.1. Hydrogel Synthesis and Its Gelation Chemistry

The general strategy of hydrogel synthesis includes cross-linking the monomers/polymers in the presence of a large volume of H_2_O [43]. The intrinsic properties of hydrogels, such as hydrophilicity and ionic/electronic conductivity, depend on the functional groups of monomers/polymers. The chemical cross-linking of the monomers/polymers can be performed either via non-covalent/physical or covalent/chemical approaches. Non-covalent cross-linking includes π−π interactions, UV/gamma radiation, and ionic/electronic/electrostatic/intermolecular interactions, whereas covalent cross-linking includes radical polymerization, condensation reactions between polymers, click chemistry reactions, and radical-mediated polymerization reactions [44]. The gelation kinetics of monomers/polymers primarily depend on the surface chemistry of the monomers/polymers, the type of cross-linking agent used, and the synthesis medium, such as the concentration of the polymer and temperature and pH of the aqueous monomer solutions. Hydrogel synthesis is performed in the aqueous medium, composed of either an acid/base/neutral solution, and hence, the resulting gels are found to have a substantial amount of surface hydrophilic functional groups that include hydroxyl, carboxylic, and amine, etc., which can interact with H_2_O [45]. The synthesized hydrogels form an excellent interconnecting network of porous nanoarchitectures with an extremely high surface area and superb electronic conductivity suitable for electrocatalysis, often post-treatment of the hydrogels via freezing, drying, or supercritical drying. The type of drying method is found to have a major influence on the morphology and porosity of the resultant electrocatalysts. After drying, the hydrogels are then subjected to thermal treatment/pyrolysis in the inert atmosphere, which can form graphitized carbons, and if the hydrogel also contains the metallic ions trapped in the polymer network, after thermal treatment, it forms metal-containing catalysts in the form of metallics/metal oxides/metal carbides/metal nitrides and atomically dispersed metal active sites, which can serve as electrocatalysts for the ORR [46]. The controlled porosity and metal active site density in the resultant catalyst depend on the carefully chosen temperature and post-treatment methods. In contrast to traditional solvothermal/hydrothermal techniques, the hydrogel-templating method is suitable for a broader spectrum of materials and precursors, which can produce interconnected micro-/nanostructures with precise doping control and enhanced chemical homogeneity at comparatively low temperatures.

#### 3.1.2. Hydrogel-Derived ORR Catalysts

To reduce the cost of ORR catalysts, recently, transition metal-based catalysts (denoted as TM-N_x_-C) have been proposed as potential electrocatalysts. Among several TM-N_x_-C catalysts, Fe-N_4_-C catalysts have been considered the most promising electrocatalysts, due to their high ORR activity from their Fe-N_4_-C geometric configuration. The most acceptable synthesis process of Fe-N_4_-C catalysts is using the metal–organic frameworks of Zn (ZIF-8), which results in microporous confined Fe-N_4_-C moieties due to their high N-content, resulting from the carbonization of a 2-methyl imidazole precursor. Though the Fe-N_4_-C catalyst derived from ZIF-8 can deliver excellent ORR activity, the microporous nature of the catalysts limits the mass transport of the reactants to the catalyst surface, which is detrimental to the catalytic activity. To control the porous nature of the catalysts, Guo et al. [47] used a hydrogel-based synthesis approach to fine tune the mesoporous nature of the HP/FeCo-NC-2 catalyst, simply by adjusting the amount of hydrogel added during the synthesis of the HP/FeCo-NC-2 catalyst. The FeCo hydrogel was synthesized by adding Fe and Co nitrate salts to the mixture of melamine and salicylic acid in aqueous solution in the presence of span-85 surfactant, and the mixture was then subjected to heating at 70 °C for 15 min to form the hydrogel. The formed gel was transferred into ice-cold water, to which 2-methyl imidazole and Zn nitrate precursors were added, which allowed the ZIF-8 to grow on the porous hydrogel template. The resulting FeCo hydrogel/ZIF-8 precursor was subjected to pyrolysis at 950 °C to obtain the HP/FeCo-NC-2 catalyst. The SEM analysis of the hydrogel-derived HP/FeCo-NC-2 catalyst showed coral-like morphology, and the HR-TEM images showed abundant honeycomb-like pores. It was observed that the quantity of the hydrogel significantly affected the porosity and pore voids on the carbon matrix. The catalyst with a smaller amount of hydrogel had reduced porosity, whereas a high hydrogel content led to significant improvements in the porosity, which helped to enhance the mass transfer of the reactants to the catalyst surface [48]. HR-TEM analysis shows no evidence of the metallic phase of Co or Fe, suggesting that they are atomically dispersed on the high-surface-area carbons. The important aspect of hydrogel-derived catalysts is the high surface area and enhanced porosity. The HP/FeCo-NC-2 catalyst exhibited a high porosity of 771 m^2^ g^−1^. The N_2_ adsorption/desorption isotherms clearly suggest that the HP/FeCo-NC-2 catalyst contains a large number of mesopores with a pore size of 4 nm, due to the hierarchical structure of the hydrogel, which guarantees the rapid mass transfer of reactants and desorption of H_2_O from active sites [49]. The ORR activity of the HP/FeCo-NC-2 catalyst is found to have similar performance to Pt/C, with a half-wave potential of 0.865 V vs. RHE.

**Figure 3:**
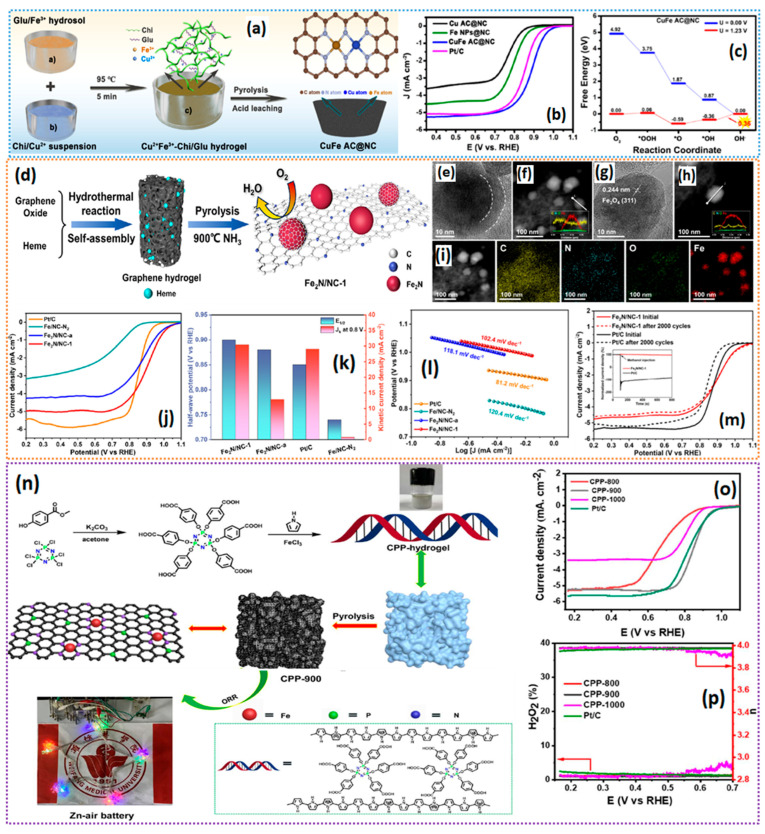
(**a**) Graphical representation of the synthesis of CuFeAc@NC catalyst. (**b**) LSV curves of various Cu AC@NC, Fe NPs@NC, CuFe AC@NC, and Pt/C catalysts. (**c**) Free energy diagram of CuFe AC@NC catalyst for ORR [Reproduced with permission from Ref. [52]]. (**d**) Graphical representation of the synthesis of Fe_2_-N/NC-1. (**e**–**i**) HR-TEM images of Fe_2_-N/NC-1 catalyst and their corresponding elemental mapping for C, N, O, and FE atoms. (**j**) LSV curves of Fe_2_-N/NC-1 and other control samples with Pt/C. (**k**) Comparison between E_1/2_ and J_k_ at 0.80 V. (**l**) Tafel slopes of Fe_2_-N/NC-1 and other control samples with Pt/C. (**m**) LSV curves of Fe_2_-N/NC-1 and Pt/C catalysts before and after 2000 cycles. Inset: the methanol tolerance test of Fe_2_-N/NC-1 and Pt/C catalysts [Reproduced with permission from Ref. [53]]. (**n**) Graphical representation of the synthesis of CPP-hydrogel and CPP-900 catalyst. The zinc–air battery illuminated by CPP-900 as cathode catalyst. (**o**) LSVcurves and (**p**) number of electrons and their corresponding peroxide yield of CPP catalysts and Pt/C [Reproduced with permission from Ref. [54]].

The atomically dispersed states of the metallic active sites maximize atomic utilization, which is crucial for electrochemical applications. Recently, dual-metal atom site-based materials have gained special interest in electrochemical reactions such as the ORR, due to the synergistic effect of electronically modifying the carbon matrix and favorable d-band center, which is suitable for the optimal adsorption energy of the ORR intermediate [50]. However, the common concern in synthesizing the dual-metal atom site-based materials is controlling their distribution on the carbon matrix. Traditional methods such as the physical mixing of the carbon, and metal–salt precursors followed by pyrolysis, often lead to the uneven distribution of metallic active sites, along with the possibility of aggregation, leading to the underutilization of the effective active surface area [51]. Most often, the distribution of the metallic sites depends on the characteristics of the carbon support, along with the surface functional groups that chelate with the metal precursors during the synthesis process. Consequently, to tackle the difficulties in synthesizing dual-metal sites, it is essential to choose a carbon support characterized by a substantial specific surface area and a high concentration of chelating groups to stabilize dual-atom sites, optimize electronic structures, and attain a precise spatial configuration of dual-atom catalysts. In this context, Wang et al. [52] proposed a chitosan–glutamic acid composite hydrogel as a sustainable catalyst, in which -NH_2_ and -OH functional groups of chitosan and -COOH and -NH_2_ groups of glutamic acid form H-bonds, and electrostatic interaction helps in chelating bonds with metallic precursors (Figure 3a). Ensuring the uniform distribution of bimetallic ions within the hydrogel matrix and effectively preventing aggregation during pyrolysis is the multi-functional fold that is guaranteed by the electrostatic interactions of the chitosan–glutamic acid composite hydrogel. The synthesis process of the chitosan–glutamic acid composite hydrogel follows the mixing of two solutions containing Glu/Fe^3+^ hydrosol and Chi/Cu^2+^ hydrosol to form a Cu^2+^/Fe^3+^/Chi/Glu hydrogel, which is then pyrolyzed to obtain a CuFe AC@NC catalyst. The obtained catalyst is found to have a 3D self-supporting structure due to the 3D network structures of Chi/Glu-coordinated Fe^3+^/Cu^2+^ ions in the hydrogel.

The 3D network structure guarantees the even distribution of Cu and Fe active sites, as observed from TEM analysis of the catalyst, where a graphene-like morphology was noted. X-ray absorption near-edge structure (XANES) analysis revealed Fe–N_4_ and Co-N_4_ moieties as ORR active sites in the CuFe AC@NC catalyst. The resulting CuFe AC@NC catalyst exhibited ORR activity on par with a commercial Pt/C catalyst with a half-wave potential of 0.887 V vs. RHE, and an average number of electrons of ~3.82–3.91 (Figure 3b). In the Zn–air battery performance analysis, the CuFe AC@NC catalyst showed excellent rate capability and specific capacitance. The density functional theory of the CuFe AC@NC catalyst modeled as two N-bridged CuN_4_ and FeN_4_ moieties (abbreviated as CuFeN_6_) suggests that the electronic density and d-band center shift to the fermi level, implying effective interfacial electron transfer between the carbon matrix composed of CuFeN_6_ and ORR intermediates [55]. The d-band center analysis of the CuFe AC@NC catalyst reveals the significant overlapping of the 3D orbitals of Cu and Fe, alongside the N 2p orbitals, suggesting a clear synergistic effect and strong interaction between CuN_4_ and FeN_4_ moieties, which is essential for the optimization of the charge transfer during the ORR catalytic process [56]. The free energy diagram of CuFe AC@NC shows a downward trend, indicating the spontaneity of the ORR, and the first electron adsorbed to the oxygen (O_2_ → *OOH) is found to be at 0.06 eV, much lower than its counterpart catalysts, suggesting the enhanced ORR kinetics (Figure 3c).

The high-temperature pyrolysis of the carbon precursor and metal salts is considered the universal strategy for synthesizing M-N_x_-C catalysts. However, it is often seen that high-temperature pyrolysis requires carefully chosen carbon support and pyrolysis conditions in order to obtain uniformly dispersed metallic nanoparticles or atomically dispersed single-atom catalysts; otherwise, it can lead to the agglomeration of nanoparticles or clustering of the atomically dispersed metallic sites [57]. To alleviate the stated problem, it is essential to mitigate nanoparticle aggregation during the pyrolysis process. In this aspect, graphene hydrogel (GH) is a fascinating material with a unique cross-linking network and spatial configuration; it has recently gained a lot of attention as a potential scaffold for building carbon-based materials with immobilized metal particles for ORR electrocatalysts. GHs are typically synthesized via a hydrothermal reaction with a graphite oxide (GO) precursor via self-assembly of the GO sheets in a 3D architecture. The 3D architecture of GH has a high surface area and porous carbon support to host nanoparticles [58]. Lei et al. [53] proposed Fe_2_N NPs synthesis via a graphene hydrogel-bridged pyrolysis strategy for the ORR. The GH–heme is pyrolyzed in an NH_3_ atmosphere to obtain the graphene supported by Fe_2_N NPs (Figure 3d). During the hydrothermal process, GO and heme moieties form a cross-linking structure via hydrogen bonds, and the interlayer spacing occupation of heme also inhibits the restacking of GO during the hydrothermal process. The TEM images of the catalysts show that Fe_2_N nanoparticles are encapsulated by carbon shells through a hydrogel-bridged strategy (Figure 3e–i). The BET surface area of the Fe_2_N/NC-1 catalyst was measured to be 216 m^2^ g^−1^, which is just sufficient for electrochemical reactions. Fe_2_N/NC-1 demonstrates a remarkable E_onset_ of 1.06 V versus the reversible hydrogen electrode (RHE, with all potentials referenced to RHE), and an E_1/2_ of 0.90 V, significantly surpassing the benchmark Pt/C (E_onset_ of 0.96 V and E_1/2_ of 0.85 V), alongside the lower taffel slope values and ideal four-electron reduction of O_2_ (n) = (3.91–3.99) in a 0.1 M KOH electrolyte (Figure 3j–m). This clearly suggests that the hydrogel-bridged strategy derived from Fe_2_N nanoparticles can significantly improve both the kinetics of the ORR and the selectivity for the four-electron pathway.

It is well accepted that MOF-derived materials offer excellent versatility to make efficient electrocatalysts made of atomically dispersed single-atom catalysts (SACs) of transition metals such as Fe-N_4_-C- and Co-N_4_-C [59]. However, the MOF-derived catalysts’ porosity is still not sufficient for the effective mass transport of gaseous O_2_ and the removal of H_2_O from the active site. Although MOF-derived catalysts excel in terms of ORR activity, stability, and mass activity, numerous researchers contend that catalysts derived from MOFs are constrained by the fact that the majority of the active sites are hidden within the carbon matrix, due to abundant micropores, thereby limiting their accessibility to reactants [60,61]. It is also believed that most of the catalytically active Fe-N_4_-C- and Co-N_4_-C moieties are hosted inside the micropore of the catalysts, and that mesopores are solely responsible for transporting the reactants to the micropores to enhance ORR activity. Therefore, it is particularly important that an ideal balance between micro- and mesopores through interconnectivity is achieved to boost the ORR kinetics and minimize the mass transfer resistance in the catalysts [62]. To tackle this, Parida et al. [63] proposed a cobalt acetylacetonate/polypyrrole (Co(acac)_3_@PPy) hydrogel precursor to obtain a catalytically active catalyst with a high density of atomically dispersed Co-N-C. The uniform distribution and dense population of Co-N-C sites are caused by the hydrophilic nature of the hydrogel, which enables easy access to the Co precursors in the polypyrrole hydrogel framework through three-dimensional channels [64,65]. The TEM measurements indicate a mesoporous structure and atomically dispersed Co atoms with no agglomeration. The uniform distribution of Co atoms results from the robust interactions of Co^3+^-N-Ppy in the hydrogen precursors, which effectively immobilize the Co atoms during pyrolysis, resulting in stable coordinated structures of Co-N-C active sites. The catalyst synthesis commences with the initial polymerization of polypyrrole in the presence of surfactant SDS, resulting in the formation of polypyrrole (ppy) hydrogel. The high porosity and interconnected pore architecture of ppy hydrogel facilitate the adsorption of Co^3+^ onto the N functionalities of ppy. Upon pyrolysis, the catalyst exhibited a high density of Co-N-C functionalities, along with excellent mesopores in the catalyst layer, which could facilitate the easier mass transport of the reactants. Surprisingly, the pore structure and interconnectivity were found to be preserved even after the pyrolysis of the Ppy-SDS hydrogel. The BET surface area of the Co-N-C catalyst was found to be in the range of 411–493 m^2^/g, along with the micro- and mesopores over a broad range. The ORR activity of the Co-N-C-0.02 catalyst presented a half-wave potential of 0.825 V for RHE, identical to that of the Pt/C catalyst.

**Figure 4:**
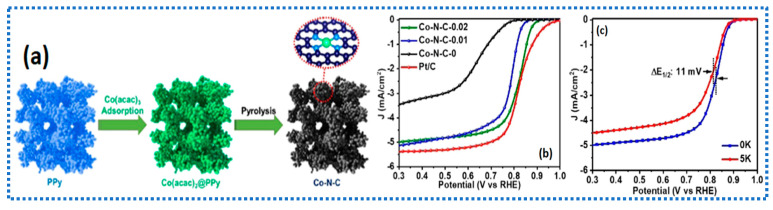

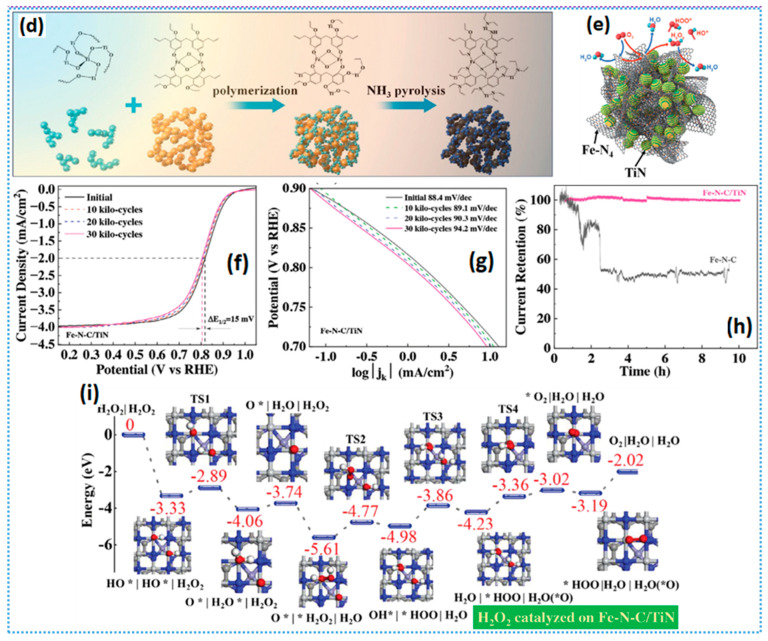
(**a**) Graphical representation of Co-N-C catalyst. (**b**) LSV curves. (**c**) Durability of Co-N-C-0.02 catalysts in O_2_-saturated 0.1M KOH solution [Reproduced with permission from Ref. [63]]. Graphical representation of (**d**) the synthesis of Fe-N-C/TiN catalyst and (**e**) TiN/Fe-N_4_ active sites stocked on carbon substrate and the peroxide mitigation via TiN. (**f**) Stability LSV curves. (**g**) Tafel slopes of Fe-N-C/TiN catalysts before and after 10 K, 20 K, and 30 K potential cycles. (**h**) Chronoamperometric response of Fe-N-C/TiN and Pt/C catalysts. (**i**) H_2_O_2_ mitigation mechanistic pathway and energies of various reaction intermediates of Fe-N-C/TiN catalyst [Reproduced with permission from Ref. [68]].

In line with the previous observations on ppy hydrogels, including the high specific area, hierarchical pore structure, and favorable dopant levels, Guo et al. [54] proposed a polypyrrole hydrogel (CPP-hydrogel) as a universal precursor for the synthesis of highly dispersed Fe-N-C active sites. The Fe-CPP-hydrogel is cross-linked with the hexa-p-(carboxyl)phenoxy cyclotriphosphazene (CP) through electrostatic and H-bonding. The resulting cross-linked composite is pyrolyzed to obtain the final N/P/Fe-tri-doped catalyst (Figure 3n). The isolation of iron species in 3D hydrogel networks led to atomically distributed Fe on the N- and P-rich carbon skeleton. In both alkaline and acidic media, the catalyst with a 3D interconnected hierarchical porous structure and large specific area has shown competitive ORR activity against commercial Pt/C. One of the best findings of this work is the BET surface area of the N/P/Fe-tri-doped catalyst, which was found to be 1002 m^2^ g^−1^, with a pore volume of 0.837 m^3^ g^−1^. To the best of our knowledge, the surface area obtained by polypyrrole hydrogels stands out to be one of the best among hydrogel-derived catalysts. Such a high surface area and pore volume guarantee the atomically dispersed state of the metallic active sites, along with the favorable mass transport of the reactants, which positively influences the ORR kinetics. Such macropores are clearly visible from the SEM analysis of the N/P/Fe-tri-doped catalyst, i.e., the CPP-900 catalyst with interconnected three-dimensional (3D) macropores over the carbon skeleton. The CPP-900 catalyst with a high surface area also exhibited excellent ORR activity, with an onset potential of 0.986 V and E_1/2_ of 0.848 V vs. RHE, n = 3.96, and 1.72% of HO_2_^−^, surprisingly lower than the values of the commercial Pt/C catalyst in the 0.1 M KOH electrolyte (Figure 3o,p); however, in 0.1 M HClO_4_, the CPP-900 catalyst shows inferior ORR activity. In a homemade Zn–air battery (ZAB), the CPP-900 catalyst could deliver a power density of 204 mW cm^−2^. Most importantly, the i-v polarization curves of the CPP-900 catalyst at the higher current density showed improved overpotential compared to those of the commercial Pt/C catalyst, which is attributed to the 3D hierarchical porous architecture present in the catalyst, which could facilitate the favorable mass transfer of reactants and quick desorption of the products during the discharge process [66]. At the current density of 10 mA cm^−2^, the ZAB with the CPP-900 catalyst lost for about ~96.5 hr with a specific capacity of 811.4 mA h g^−1^ Zn, which is about 99% of its theoretical capacity. Furthermore, the ZAB utilizing the CPP-900 catalyst exhibits remarkable durability over approximately 1000 repetitive charge–discharge cycles at a current density of 10 mA cm^−2^, corroborating the high stability conclusions derived from the ORR for ZAB applications. In addition to ORR catalysis, nanostructured framework materials originating from 3D hydrogels can function as bifunctional electrocatalysts for both the ORR and OER. In a significant study by Liming Dai et al. [67], they synthesized a 3D hydrogel from the polymerization of the aniline monomers in the presence of phytic acid as a cross-linking agent to produce a PANi hydrogel. The obtained PANi–phytic acid hydrogel was then freeze-dried into an aerogel and, with subsequent pyrolysis, formed a 3D N- and P-doped highly connected hierarchical network of mesoporous carbon (NPMC) (Figure 4a). In addition to the 3D porous network, the NPMC catalyst was also found to have edge-like graphitic structures. Most interestingly, the NPMC-1000 possessed an extraordinary BET surface area of 1663 m^2^ g^−1^, which is incredible and much larger than that of the traditional template-based porous catalyst synthesis routes. The NPMC-1000 catalyst with a 3D mesoporous network with P and N dopant exhibited an onset potential of 0.94 V and a half-wave potential of 0.85 V vs. RHE, and its n was determined to be 4, with a hydro-peroxide yield of ~8% (Figure 4b,c). The DFT analysis revealed that the 3D porous-structured NPMC-1000 overpotentials for the ORR and OER were about 0.44 and 0.39 V, respectively, which are lower than those of the commercial standard catalysts, such as Pt/C (0.45 V) and IrO_2_ (0.42 V), suggesting that P- and N-doped 3D carbon could outperform the conventional standard catalysts.

In short conclusion, the ORR activity of the hydrogel-derived catalysts was analogous to that of the commercial Pt/C catalyst, and in certain instances, it surpassed it. The primary benefit of hydrogel-derived catalysts is their extensive surface area and three-dimensional porous architecture, which alleviates the mass transfer constraints present in commercial Vulcan carbons. The hydrophilic characteristics of the hydrogels promote the diffusion of metallic precursors, assuring their uniform distribution during pyrolysis, which leads to atomically distributed catalysts. Hydrogel-derived catalysts can also be utilized to optimize the equilibrium between micro- and mesopores, which is crucial for accommodating the micropore-M-N_x_-C active sites that receive the read tabs from the mesopores and macropores. Among the various hydrogels, the polypyrrole hydrogels (1002 m^2^ g^−1^) and polyaniline–phytic acid (1663 m^2^ g^−1^)-based hydrogels appear to excel in providing a superior surface area and three-dimensional porous structures, which contribute to improved ORR activity, stability, and power density performance.

## 4. Aerogels

Aerogels are synthetic solids with meso- and macropores with diameters of up to a few hundred nanometers, derived from the sol–gel synthesis process with a highly porous, 3D solid network in which gas occupies 90–99% of the entire volume of the gel, used in heterogeneous catalysis and other applications such as microwave absorption and electromagnetic wave adsorption [69,70]. The aerogels result from the replacement of the liquid solvent by the gaseous component, which helps with keeping the 3D porous network intact, resulting in an ultra-low density (0.003 g cm^−3^), a high surface area (>1000 m^2^ g^−1^), and excellent continuous porosity, desirable for the electrocatalytic applications [71]. The aerogel’s derived carbons have been traditionally used as catalyst supports for fuel cells and other electrocatalytic reactions to mitigate the Pt nanoparticle agglomerations and highly interconnected porosity, leading to the enhanced mass transport of the reactants to the active site [72,73]. The aerogel catalysts are generally synthesized by a two-step process: (i) the synthesis of the gel via the sol–gel method and (ii) the drying of the gel at under specified conditions.

### 4.1. The Sol–Gel Synthesis of the Gel

The aerogels’ synthesis follows the formation of a monolith gel from the precursors. The gel is formed from the condensation or polymerization of molecular precursors such as silica, metal oxides, and certain organic polymers. The gels are classified based on the type of precursor used. For example, with metal alkoxides, M(OR)n undergoes a hydrolysis–condensation reaction, resulting in the formation of M-O-M bonds, which are called “metal-oxide aerogels” [74]. The “Organic aerogels” are formed from the organic polymer precursor with strong covalent (-C-C-). “Carbon aerogels” are formed from the hydrolysis–condensation reaction between resorcinol and formaldehyde [75]. In addition to these conventional synthesis routes, there has been a recent surge in interest in the use of graphene, CNTs, and nanofibers as precursors for carbon aerogels, as a result of their exceptional surface area, chemical stability, and electrical conductivity [76,77].

### 4.2. The Key to 3D Gel Structure—Gelation

A stable combination of particular molecules or nanocolloids is called a “sol”. The primary objectives of the gelation process are to make the sol unstable in a way that can be controlled and to connect the precursors so that the solid network forms from the liquid solvent. Acids, bases, and proton scavengers are typically the catalysts for gel formations that use molecular precursors, and the concentration of the precursors or catalyzing agents regulates the gelation kinetics [78]. The creation of homogenous gels is made possible by the use of molecular precursors, which make it simple to mix two or more precursors uniformly at the atomic level. The rate at which the monomers polymerize determines the gelation kinetics in the case of organic gels [79]. Carbon aerogels treated with high temperatures yield highly porous, electrically conducting carbons with a large surface area and an intact porous network. However, structural precursors like oxidized or surface-functionalized graphene or CNTs are also used to form the sols, which are typically stabilized using surfactants, in contrast to molecular precursors. In the case of a structural precursor, the molecular precursors self-assemble into a randomly connected network during the gelation process [80]. The hydrothermal synthesis process is used to carry out the majority of structural precursor-mediated gel syntheses. The use of cross-linkers is also widely accepted as a feasible alternative to self-assembly-mediated gel formation, whereby cross-linkers create electrostatic or covalent/hydrogen bonds to cause gelation.

### 4.3. Obtaining Aerogels from Wet Gels and Carbonization

Gels are always accompanied by large amounts of solvents trapped in the porous monolith; the elimination of the solvent by drying the gel is a critical and final step in obtaining the aerogels. When the solvent-containing gels are dried via conventional drying (ambient/hot-air oven drying), the resulting powders are called “xerogels” and the drying process is accompanied by the disintegration of and decline in the intrinsic 3D porous network of the gel. In order to achieve aerogels with the intrinsic 3D porous network retained in the final product, freeze-drying or supercritical drying is generally employed [81]. The liquid CO_2_ is replaced by solvent molecules in the gel, simultaneously increasing the pressure and temperature to bring the system to a critical point. This is followed by the elimination of CO_2_ by lowering the pressure while maintaining the constant temperature. The benefit of supercritical drying is that it significantly reduces the solvent’s destructive effect on the surface tension, maintaining the gels’ three-dimensional fine structure, preventing gel shrinkage, and keeping their porous structure intact. On the other hand, freeze-drying involves the freezing of the solvent crystals and the sublimation of the frozen solvent [82]. In contrast to supercritical drying, there are possibilities of damage to the porous structures caused by the crystallization of the frozen solvent inside the porous structures, resulting in slightly lower-surface-area powders. However, due to its economic feasibility, freeze-drying is still considered a popular method for producing aerogels [83]. The final step is obtaining carbon powders from the dried aerogel via pyrolysis in an inert atmosphere, at temperatures higher than >600 to 900 °C, to be used as electrocatalysts.

### 4.4. Aerogel-Derived ORR Catalysts

Aerogels containing heteroatoms such as N, S, and P, etc., along with transition metals, are attractive catalysts for the ORR due to their high surface area, hierarchical porosity, presence of heteroatom atoms that modulate the electronic structure and enhance O_2_ adsorption, and presence of highly active Fe-N_4_ or Co-N_4_ active sites. Luo et al. [68] synthesized a highly graphitized, high-surface-area, porous aerogel-derived Fe-N-C/TiN catalyst via supercritical drying that can deliver high ORR activity (Figure 4d,e). Most importantly, in this study, a novel TiN radical scavenger was introduced into fuel cell electrocatalysis. The reaction between resorcinol–formaldehyde, TiO_2_ sol, and Fe-doped resorcinol–formaldehyde polymer leads to generating a new hydrogel network with a TiO_2_-doped polymer skeleton. The resulting hydrogel network is subjected to high-temperature pyrolysis in the presence of NH_3_, resulting in the Fe-N-C/TiN catalyst. NH_3_ treatment is found to be an important source of TiN formation from the TiO_2_ phase and the generation of Fe-N-C active sites. The XRD analysis revealed the TiN phase and graphitic carbon diffraction peaks; the absence of any type of Fe phase such as metallic/carbide/nitride suggests that Fe atoms are possibly in the atomically dispersed state. The observed TiN particle size is 15 nm, with a nanosized skeleton and interpenetrated network of TiN and carbon heterostructures. No visible aggregates of Fe are seen, and hence, it can be concluded that Fe active sites are distributed in an atomically dispersed state, implying the superiority of sol–gel synthesis processes in generating porous Fe-N-C/TN heterostructured catalysts. The Fe-N-C/TN catalyst in the 0.1 M HClO_4_ electrolyte exhibited extraordinary stability, with a loss of just 15 mV in its half-wave potential after 30,000 potential cycles, with a peroxide yield of below 3%, whereas the Fe-N-C catalyst possesses a peroxide yield of >10% (Figure 4f–h). This RRDE measurement clearly hints that the TiN acts as a potential radical scavenger. The enhanced durability of Fe-N-C/TN can be seen from the only slight reduction in the double layer capacitance values from 42.8 to 42.1 μF cm^−2^. The Fe-N-C/TN catalyst had a H_2_-O_2_ fuel cell performance of 1050 mW cm^−2^, with a back pressure of 200 kPa. The Fe-N-C/TN catalyst’s performance deteriorated to 765 and 614 mW cm^−2^ for 10,000 and 30,000 potential cycles, respectively. The extraordinary stability of the Fe-N-C/TN catalyst is due to the corrosion resistance and radical scavenging of the TiN. To elucidate the radical scavenging mechanism of the aerogel-synthesized Fe-N-C/TN catalyst, EPR measurements were conducted. The EPR signals for the Fe-N-C/TN catalyst were found to be significantly lower than those of the Fe-N-C catalyst. DFT analysis further revealed that the adsorption energy (−ΔG) of H_2_O_2_ and free radicals was more negative for the Fe-N-C/TN catalyst than the Fe-N-C catalyst, implying that H_2_O_2_ and free radicals adsorb preferentially on the Fe-N-C/TN catalyst. Furthermore, the decomposition of H_2_O_2_ into *OH is kinetically faster on the Fe-N-C/TN catalyst than on the Fe-N-C catalyst, due to the increased negative energy change of −3.33 eV compared to −2.41 eV for the Fe-N-C catalyst (Figure 4i). The experimental and theoretical investigation clearly established the radical scaling ability of TiN and that it is the primary cause of the excellent stability of the Fe-N-C/TN catalyst in the 0.1 M HClO_4_ electrolyte.

**Figure 5:**
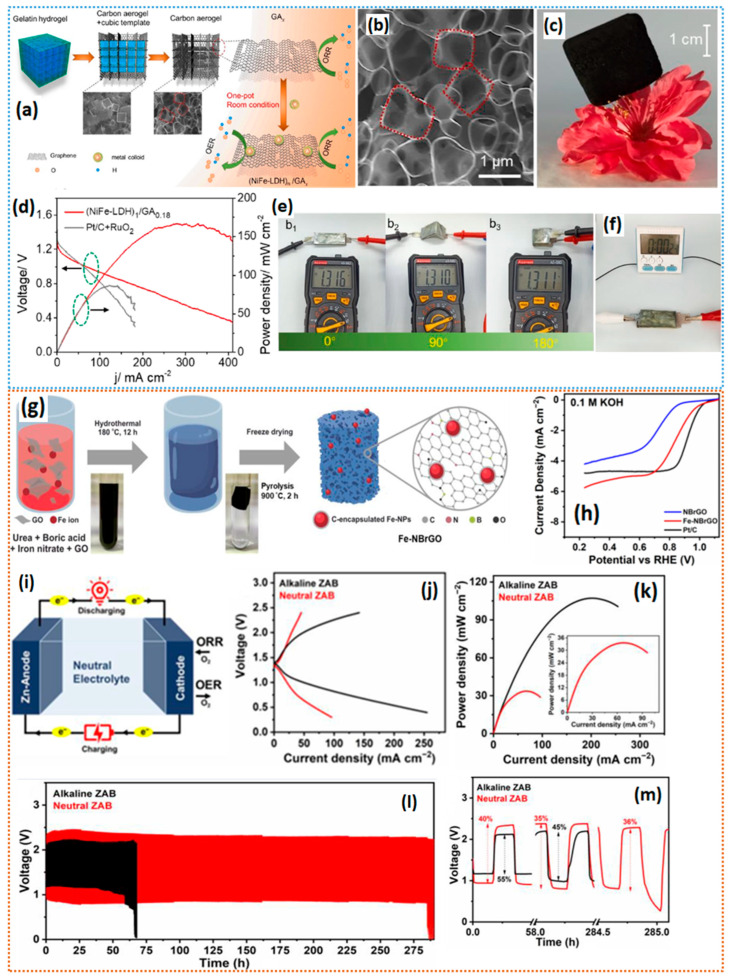
(**a**) Graphical representation of synthesis and (**b**) SEM images of (NiFe-LDH)_n_/GA_x_ catalyst. (**c**) Picture of a part of GA catalyst placed on peach blossom flower. (**d**) Zn–air battery polarization curves of (NiFe-LDH)_n_/GA_x_ and Pt/C + RuO_2_ catalysts. (**e**) OCP measurements of solid-state Zn–air battery with different bending angles of b1 = 0°, b2 = 90°, and b3 = 180°. (**f**) Picture of solid-state Zn–air battery with electronic clock powered by solid-state Zn–air battery with NiFe(NiFe-LDH)_n_/GA_0.18_ as cathode catalyst [Reproduced with permission from Ref. [85]]. (**g**) Graphical representation of the synthesis. (**h**) LSV curves of Fe-NBrGO catalyst. (**i**) General schematic of working principle of rechargeable Zn–air battery. (**j**) Discharge–charge–charge curves. (**k**) Polarization curves. (**l**) Stability curves. (**m**) Voltaic efficiency of ZAB with Fe-NBrGO catalyst in neutral and alkaline conditions [Reproduced with permission from Ref. [87], open access].

The catalytic activity of the electrocatalysts primarily depends on the density and extent of active site utilization [84]. In this context, 3D carbon aerogels are particularly attractive due to their abundant channels of porosity and high surface area, which helps to homogeneously distribute the metallic active sites, and the highly interconnected pores help in alleviating the mass transfer limitations. Integrating the graphene sheets into the carbon aerogels can further maximize the accessible surface area. Li et al. [85] proposed a bottom-up strategy for the fabrication of carbon–aerogel–graphene heterostructures loaded with NiFe-LDH colloids. First, the high-temperature pyrolysis of the gelatin hydrogels produces the carbon aerogels onto which the colloids of NiFe-LDH are adsorbed, by immersing the aerogels in a NiFe-LDH solution [86], which, upon room temperature reduction, resulted in the NiFe-LDH_n_/GA*_x_.* SEM and TEM measurements reveal a highly porous carbon network (Figure 5a–c). The NiFe-LDH_n_/GA_0.18_ catalyst showed a high half-wave potential of 0.840 V, which is slightly higher than that of the Pt/C catalyst—0.831 V. The obtained high ORR activity is attributed to the excellent electronic conductivity, high surface area, densely populated Ni and Fe coordinated-N active sites, and exposure of highly active graphitic edge sites for ORR. In a zinc–air battery setup, the NiFe-LDH_n_/GA_0.18_ catalyst delivered higher performance than the commercial Pt/C-RuO_2_ catalysts. In addition, in a solid-state Zn–air battery setup, the NiFe-LDH_n_/GA_0.18_ catalyst showed consistent OCV values at different bending angles and in different shock wave conditions (Figure 5d–f), indicating that the NiFe-LDH_n_/GA_0.18_ catalyst could be ideal for flexible electronic applications. Irmawati et al. [87] synthesized an Fe-decorated N- and B-co-doped reduced graphene oxide aerogel for the enhanced ORR activity in alkaline electrolytes. The synthesis process follows the hydrothermal treatment of GO and the precursors that include urea, boric acid, and Fe (NO_3_)_2_, followed by pyrolysis at 900 °C. The XRD analysis shows the multiphase Fe particles with Fe, Fe_3_C, and Fe_3_O_4_, and TEM analysis shows the agglomerated particles of Fe_3_C. The resulting Fe-NBrGO catalyst showed a half-wave potential of 0.826 V vs. RHE (Figure 5g,h). Fe-NBrGO enables the production of a neutral ZAB, with a 34 mW cm^−2^ peak power density and that remains stable for a 284 h (~852 cycles) cycling test, whereas alkaline ZAB with Fe-NBrGO exhibits a promising peak power density of 107 mW cm^−2^, comparable to previous studies in the range of 123 mW cm^−2^, showing a more than three times longer battery cycle life than neutral electrolytes. Neutral ZAB with Fe-NBrGO exhibits up to 284 h of cycling stability, surpassing M-N/C catalysts with 70–208 h of cyclability. The neutral ZAB’s high round-trip stability is confirmed by its voltaic efficiency, calculated by dividing the discharging potential by the charging potential (Figure 5i–m). Bai et al. [88] synthesized a PdCu alloy-decorated N-doped carbon aerogel catalyst for ORR via sol–gel and freeze-drying methods. The SEM and TEM images show excellent porosity and crystalline PdCu nanoparticles. The aerogel-derived PdCu@NC catalyst showed significantly improved ORR activity, with a half-wave potential of 0.925 V vs. RHE, much higher than that of the Pt/C catalyst.

Cytochrome C oxidase is a nature-inspired enzyme-based ORR catalyst that contains Fe and Co porphyrins coordinated with organic histidine ligands [89]. Though the cytochrome C oxidase carried out excellent oxygen reduction at the cellular level, in fuel cell harsh atmospheric conditions, the activity is generally underrated due to the bulky nature of the enzyme and the low active site density [90]. One of the popular approaches to increasing the activity of this enzyme is by immobilizing the carbon support [91]. Persky et al. [92] synthesized an aerogel-based Fe-porphyrin that was covalently attached to Cu-corrole structures and a FeCu porphyrrole aerogel. The FeCu porphyrrole aerogel was synthesized via a traditional Schiff base reaction that resulted in a FeCu porphyrrole aerogel that was dried via CO_2_-mediated supercritical drying to obtain the final precursor, which, upon high-temperature thermal treatment at 800 °C, yielded the conductive FeCu catalyst. The SEM images of the aerogel catalyst showed semispherical aggregates that were interconnected with large void macropores. The pore size distribution analysis revealed the presence of micropores with a pore size of 1–1.5 nm, along with abundant mesopores that help in the enhanced mass transport of oxygen in the catalyst layer [93], while TEM measurements show the atomically dispersed Fe and Cu atoms on the graphitized carbon support. In alkaline electrolytes, the FeCu porphyrrole aerogel catalyst exhibited an ORR onset potential of 0.94 V and a half-wave potential of 0.80 V vs. RHE. Interestingly, the HT-FeCu porphyrrole aerogel catalyst also showed excellent stability, with a loss of just 16% in the relative current in a chronoamperometric test for 24 h at 0.6 V. What is more interesting is that the HT-porphyrrole aerogel catalyst not only showed admirable ORR activity, but also delivered a power density of 420 and 510 mw cm^−2^ in a realistic AEMFC in a membrane electrode assembly configuration.

This study opens up a new avenue for utilizing nature-inspired materials for ORRs synthesized via an aerogel route. In a similar trend, Zion et al. [94] reported an atomically dispersed porphyrin-based catalyst (FeP aerogel) with excellent ORR activity and fuel cell performance in AEMFCs. The FeP aerogel was synthesized using Fe^2+^ and porphyrin ligand (5,10,15,20-(tetra-4-aminophenyl)), which was polymerized and cross-linked using terephthalaldehyde as a cross-linking agent. Upon a cross-linking reaction for 24 h, a gel was formed, which was then dried via supercritical drying with liquid CO_2_ and pyrolyzed at 800 °C. It is important to identify that the ORR activity of the FeP catalyst was highly dependent on the heat treatment temperature [95,96]. It is observed that the ORR activity steadily increased with the increase in temperature from 600 °C to 800 °C, and then declined. The HT800-FeP catalyst showed the optimum ORR activity of 0.96 V onset and 0.86 V vs. RHE half-wave potentials, and most importantly, the yield of HO_2_^−^ % was found to be <1%. In H_2_-O_2_-based AEMFCs, the HT800-FeP catalyst delivered a maximum power density of 580 mW cm^−2^, with a catalyst loading of 1.25 mg cm^−2^. The excellent ORR activity and fuel cell performance of the HT800-FeP catalyst are credited to the high density of Fe-Nx active sites.

Polyaniline-based hydrogels and the corresponding aerogels remain some of the most important precursors for the synthesis of highly active M-N_x_-type catalysts, due to the presence of N on the back of aniline that coordinates with transition metal atoms via H-bonding. In view of this, Tang et al. [97] synthesized a novel ferrocene–phosphorus-rich porous polyaniline (P-PANi) gel-derived Fe-N/P/C-850 catalyst with ORR activity similar to that of the Pt/C (20 wt.%) in 0.1 M KOH electrolytes. The aniline and phytic acid mixture was added to the ammonium persulphate solution to obtain the green-blue colored gel, which was subsequently freeze-dried and pyrolyzed to obtain the Fe-N/P/C-850 catalyst with a surface area of 615 m^2^ g^−1^. The Fe-N/P/C-850 catalyst displayed a high ORR onset potential and half-wave potential of 1.06 and 0.86 V vs. RHE, higher than those of the standard Pt/C catalyst, with 1.01 and 0.84 V vs. RHE, respectively. In addition, the Fe-based PANi catalysts and Ni-based catalysts have recently been given higher importance, due to their unique ability to balance the ORR and OER reaction kinetics, a major obstacle in rechargeable zinc–air batteries. Subsequently, the Fe-Ni-based catalysts have received greater attention for their ORRs and OERs [98,99]. The traditional ORR and OER active sites in such catalysts are generally ascribed to the Fe-N_4_ and Ni-N_4_ bonding configurations in the catalysts. However, recently, Li et al. [100] proposed a new type of bifunctional active site composed of N_4_–Fe–O_x_–Fe–N_4_ moieties that are strikingly different from the traditional M-N_x_ active sites. According to the authors, the introduction of the axial-bonded O atoms can significantly alter the electronic structure of the active sites. The authors utilized an aerogel synthesis strategy to incorporate such N_4_–Fe–O_x_–Fe–N_4_ moieties into the catalyst matrix. Benefiting from the strong chelation effect of tannic acid and polyaniline to Fe^3+^ ions, a hydrogel was synthesized in the presence of ammonium persulphates to obtain the PANI-TA-Fe hydrogels, which were then freeze-dried and heat-treated to obtain the Fe-Ni ANC@NSCA catalyst. The morphological analysis of the Fe-Ni ANC@NSCA catalyst revealed a 3D framework composed of densely packed 2D nano carbon sheets, with a visible microporous 3D network. BET analysis and pore size distribution assessment indicated the existence of macro-, meso-, and micropores within the catalyst layer. The existence of micro-/meso-/macroporous structures in carbon catalysts can enhance their catalytic activity and durability by reducing ionic/electronic diffusion distances and facilitating efficient mass transport channels during the electrocatalytic process [101]. The ORR activity of the Fe-Ni ANC@NSCA catalyst was found to be much higher than that of the commercial catalyst, with a half-wave potential of about 0.891 V, whereas the Pt/C catalyst displayed a half-wave potential of 0.876 V vs. RHE. In addition, the Fe-Ni ANC@NSCA catalyst performed an ORR with a nearly ideal four-electron reduction of O_2_ to OH^−^, and had excellent stability, with a loss of just 24 mV in the half-wave potential for 10,000 potential cycles. In a rechargeable Zn–air battery configuration, the Fe-Ni ANC@NSCA catalyst delivered a power density of 140 mW cm^−2^, specific capacity of 750 mA h g^−1^, and the overall potential gap of the ORR and OER (ΔE_gap_ = 1.05 V) was much lower than that of the commercial Pt/C catalyst, with ΔE_gap_ = 1.42 V. In addition, the Fe-Ni ANC@NSCA catalyst displayed excellent stability at the current density of 5 mA cm^−2^, with a negligible loss in the voltage both in the charge and discharge segments.

John B. Goodenough et al. [102], the Nobel Prize winner and his co-workers, developed a robust 3D porous FeCo/N-DNC catalyst using a novel cyanometallate cross-linked chitosan/graphene oxide dual-cross-linked hydrogel as the precursor. The hydrogel was synthesized via the polymerization of a mixture of K_4_Fe(CN)_6_, K_3_Co(CN)_6_, a natural polymer chitosan, and a 2D graphene oxide sheet to form a K_4_Fe(CN)_6_/K_3_Co(CN)_6_-CS-GO hydrogels. The precursor hydrogen is termed a dual-network gel due to the presence of two kinds of bonds in the precursor hydrogel. The chitosan -NH_2_ groups interacted with Fe and Co ions, and the -OH groups interacted with the cyanometallates. It is to be noted that the chitosan solution turned into gel only when cyanometallates were added, implying the role of cyanometallates in the gelation process. The second type of interaction is between the negatively charged oxygen functional groups of GO and the protonated -NH_2_ groups of chitosan via electrostatic interactions. A very homogeneous and strong dual-network hydrogel is anticipated as a result of two of these bonding interactions. The trapped solvent from the hydrogel was removed via freeze-drying, and the K_4_Fe(CN)_6_/K_3_Co(CN)_6_-CS-GO hydrogel was subjected to pyrolysis, which produced the 3D N-doped carbon-encapsulated FeCo particles. In addition to confirming the homogeneous distribution of FeCo nanoparticles of around 45 nm in size from the EDX line scanning profile and EDX mapping analysis, the morphological analysis of the FeCo/N-DNC catalyst reveals a highly 3D interconnected porous configuration, with continuous pores in the sub-micrometer. The FeCo/N-DNC catalyst possesses a micro- and mesoporous structure with a BET surface area of 260 m^2^ g^−1^. In electrocatalytic activity measurements, the FeCo/N-DNC catalyst exhibited an onset potential of 0.89 V and a half-wave potential of 0.81 V vs. RHE in the 0.1 M KOH electrolyte. The improved ORR activity of FeCo/N-DNC aerogels, in comparison to monometallic samples, may primarily result from the alloying effect of Fe and Co, as demonstrated in other bimetallic alloys, due to the synergistic effect of Fe and Co on O_2_ adsorption and its reduction. It is also vital that FeCo nanoparticles are in a heterogeneous structure with a strong anchoring effect, contributing to the enhanced electron and ion conductivity to the ORR active site. This strong coupling effect not only enhances the ORR activity, but also improves the number of electrons transferred in the ORR (n = 3.92), as well as stability, as it can be seen that FeCo/N-DNC catalysts could retain the relative currents of ~80% after a 10,000 s *i-t* test. In a rechargeable Zn–air battery setup, the FeCo/N-DNC catalyst could deliver a power density of 115 mW cm^−2^, a higher specific capacity (804 mA h g Zn^−1^), and energy density (988 W h kg Zn^−1^) at 5 mA cm^−2^. In the charge/discharge performance, the FeCo/N-DNC showed excellent stability. The enhanced bifunctional activity of the FeCo/N-DNC catalyst is credited to the (i) 3D interconnected porous architecture that favors the diffusion of ions/reactants during electrochemical ORR/OER reactions, (ii) the N-doped carbon layer on the surface of FeCo nanoparticles that improves the electron transfer from the N-doped carbon to the FeCo active sites, and (iii) the mitigation of FeCo nanoparticle dissolution and agglomeration, resulting in improved stability.

Fu et al. [103] synthesized Pd_3_Pb nanoparticles supported on 3D interconnected rGO-CNTs aerogels with the possibility of large-scale synthesis, with PVA as a cross-linking agent. Hydrogels composed of PVA-cross-linked GO-CNTs make it possible to efficiently collect highly active Pd_3_Pb nanoparticles following pyrolysis, without the particles adhering to or peeling off the carbon support as they undergo the electrochemical reaction. The Pd_3_Pb/rGO-CNTs catalyst’s synthesis starts from the sol–gel polymerization of oxidized CNTs, r-GO, PVA polymer, and Pd and Pb ionic metal precursors (M^n+^). Ultra-sonically mixed GO-CNTs with PVA-Mn^+^ aqueous solutions produced a stable black hydrogel. GO’s amphiphilic character and π−π interactions help to enable homogeneous CNT dispersion on GO surfaces. Hydrogen bonding between oxygenated groups on GO and carbonylated CNT and PVA hydroxyl groups drives hydrogel network formation. Furthermore, stabilizing the hybrid hydrogel structure are Mn^+^ ions’ electrostatic and coordination interactions with GO and CNTs.

The SEM and TEM measurements of the Pd_3_Pb/rGO-CNTs show the extraordinary 3D interconnected nanosheets of graphene and CNTs forming heterostructures. The 3D interconnected structure is formed by the randomly oriented wrinkled graphene sheets entwined with CNTs. These interconnected heterostructures of rGO-CNTs enhance the robustness of the catalyst. In addition, the porous nature of Pd_3_Pb/rGO-CNTs exposes abundant active sites and accessibility to the inner parts of the electrocatalyst. In the electrocatalytic performance evaluation, the Pd_3_Pb/rGO-CNT catalyst delivered an ORR half-wave potential of 0.862 V, with 3.84 electrons transferred per O_2_ molecule and a HO_2_^−^ yield of 12.3%. The enhanced ORR activity of the Pd_3_Pb/rGO-CNT catalyst is ascribed to an ordered intermetallic phase because of the ordered active sites, the alteration of the electron configuration, and the modification of the Pd−Pd bond distance. In addition, a robust combination of Pd_3_Pb particles and 3D porous graphitized rGO-CNT aerogels provides superior electrical contact with the outside circuit and facilitates the easy diffusion of active species to and from the ordered Pd_3_Pb particles.

**Figure 6:**
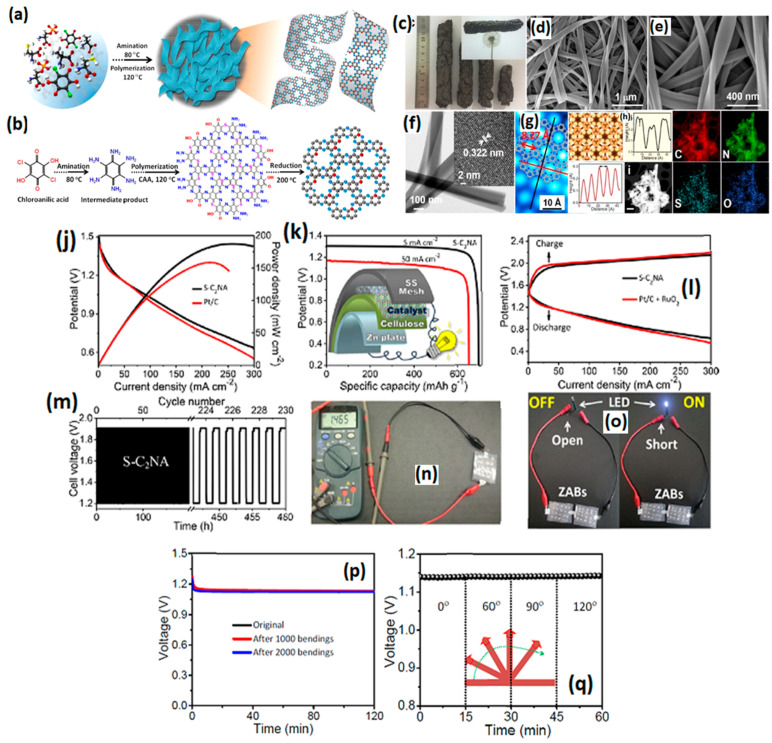
(**a**) Graphical representation of the S-C_2_NA catalyst, (**b**) reaction mechanisms of formation of COFs, (**c**) optical pictures of lightweight S-C_2_NA catalyst, (**d**,**e**) SEM, and (**f**) TEM images of the S-C_2_NA catalyst. Inset: HRTEM image of the S-C_2_NA catalysts. (**g**) Inverted atomic STEM image of S-C_2_NA catalysts on Cu (1 1 1) and the (**h**) topographic height profile. (**i**) STEM images and their corresponding elemental mapping images for C, N, S, and O solid-state Zn–air battery. (**j**) Performance curves, (**k**) specific capacity curves, (**l**) charge/discharge curves, (**m**) stability curves, (**n**) OCV optical picture, and (**o**) practical demonstration by illuminating the LED with Zn–air batteries connected in series. Discharge plots for flexible ZABs at 10 mA cm^−2^ as a function of (**p**) bending times and (**q**) bending angles [Reproduced with permission from Ref. [106], open access].

The synthesis of aerogels from hydrogels by substituting water with air, resulting in continuous porous structures utilizing COFs as building blocks, is of considerable interest, due to their three-dimensional porous network, elevated surface area, substantial pore volumes, and low density. Although the fundamental components of aerogels, including carbon nanotubes, cellulose fibers, and graphene, have been investigated, they exhibit a deficiency in precise control over porosity [104,105]. In this regard, Shinde et al. [106] proposed a COF-derived aerogel as ORR and OER catalysts aiming to improve the rechargeable capacity of the Zn–air battery. A two-step sulfur-doped holey C_2_N catalyst was synthesized by aminating the chloroanilic acid to manufacture hexa-aminobenzene complex, which was then polymerized by L-alanine and L-cysteine to construct π-conjugated polymeric reinforced C-N structures to finally obtain the COFs (Figure 6a,b). The resulting COFs are then freeze-dried and pyrolyzed to obtain the final catalyst. The SEM images revealed a sponge-like holey morphology, and the resulting aerogels also exhibited a low density (64 mg/m^3^). The TEM measurements show a nanoribbon morphology with a twisted and interweaved 3D network and a homogeneously distributed holey structure with hexagonal arrays (Figure 6c–g). The interstitial space between the nanoribbon structures provides abundant highways to ionic diffusion during the ORR, and hence facilitates rapid electrochemical kinetics and reduced mass transfer resistance [107]. The BET surface area of the catalysts revealed an extraordinary surface area of 1943 m^2^ g^−1^, a pore size in the range of micro/mesoporous structures with abundant peaks at 3 nm, and a pore volume of 1.56 cm^3^ g^−1^. To the best of our knowledge, the S-C_2_NA catalyst synthesized via the COFs presented in this study stands out the best in terms of the highest BET surface area achieved. In an RDE study, the S-C_2_NA catalyst exhibited improved ORR kinetics, surpassing the commercial Pt/C with a half-wave potential of 0.88 V vs. 0.85 V of Pt/C. The S-C_2_NA catalyst’s Zn–air battery performance has been obtained for both liquid- and solid-based electrolytes. In a liquid electrolyte-based Zn–air battery, the S-C_2_NA catalyst exhibited an OCV of 1.49 V, a power density of 209 mW cm^−2^, and excellent stability over 20 h in galvanostatic discharge conditions. Upon aiming for the application of the S-C_2_NA catalyst in flexible and wearable electronic technologies, a solid-state ZAB was constructed, which showed an OCV of 1.47 V and a powder density of 187 mW cm^−2^. The specific capacities and corresponding energy densities of the S-C_2_NA battery were 695 and 653 mA h g^−1^, and 862 and 805 Wh kg^−1^, when normalized to the mass of zinc, at current densities of 5 and 50 mA cm^−2^, respectively. In addition, the S-C_2_NA catalyst showed extraordinary stability for over 460 h of continuous operation, with almost no loss in charge/discharge potentials, suggesting S-C_2_NA catalysts’ excellent robustness. In addition, the solid-state Zn–air battery also exhibited similar performance at different bending angles, suggesting its readiness in commercial applications (Figure 6j–q).

## 5. Xerogel-Derived Catalyst

Xerogels are solid materials that combine porous solid network catalysts with dried hydrogel precursors at ambient or mild temperatures. Unlike aerogels, where the hydrogel precursor is dried under supercritical drying or freeze-drying conditions, the hydrogels are usually dried below <100 °C in a hot-air oven. One limitation of xerogels is the partial collapse of their porous network during the drying process, which prevents the full retention of the original structure from the parent hydrogel to the final catalyst. The xerogels obtained through ambient drying are then subjected to pyrolysis to obtain the final catalyst composed of metal oxides/metal carbides, etc., along with hetero-atom-doped carbons, resulting from the decomposition and doping of the precursors [108,109]. The precursor gels are usually obtained via the sol–gel synthesis route with or without cross-linking agents. The sol–gel synthesis process also includes the integration of structural carbons such as graphene/CNT/ZIFs, etc. Xerogel-derived catalysts, in general, possess a sufficient surface area, hierarchical porosity, and graphitic carbons resulting from the carbonization of the precursors [110]. The following section details the electrocatalyst xerogel-derived catalyst for the ORR.

Transition metal oxides, especially manganese oxides, have attracted great interest among non-Pt-based catalysts because of their low cost, the simplicity of redox transitions between several Mn oxidation states (Mn^2+^ ↔ Mn^3+^ ↔ Mn^4+^), and their great electrochemical stability [111]. Mn-based catalysts specifically help to reduce H_2_O_2_-associated disadvantages, since the Mn^3+^/Mn^4+^ redox couple is known to have a good ^•^OH scavenging capacity, thus improving the stability of Mn-based catalysts [112]. Although MnO_x_-based catalysts show encouraging ORR activity, their intrinsically poor electronic conductivity and tendency to aggregate under electrochemical conditions draw attention to the need for creative synthesis techniques. One of the widely accepted strategies is immobilizing the MnO_x_ on the high-surface-area carbons that can provide anchoring sites and enhance the electron transfer between the carbon and MnO_x_ nanoparticles.

Hao et al. [113] synthesized MnO_x_ nanoparticles supported on the N-doped carbon derived from sustainable waste coffee grounds. The sol–gel process was used to synthesize the MnO gel, which was dried overnight at 70 °C to obtain the dried gel, which was then subjected to heat treatment at 900 °C to obtain the MnO nanoparticles. In the synthesis process of MnO/N-doped carbon, the N-doped carbon was added to the sol–gel solution to obtain a dark monolith gel. The gel was then subjected to pyrolysis to obtain the MnO/M-doped carbon catalysts. The XRD analysis shows the crystalline nanoparticles of MnO deposited on the N-doped carbons with a particle size of 33 nm. The xerogel-derived MnO/N-CC-2-900-2 catalyst exhibited an acceptable surface area of 259 m^2^ g^−1^ and a mesoporosity of 3.28 nm. The SEM and TEM measurements show uniformly deposited MnO nanoparticles on thick graphene-like nanosheets, as evidenced from EDAX elemental mapping. The XPS analysis shows the Mn-O-Mn and Mn-OH active sites that are responsible for ORR activity. A systematic assessment of the effect of heat treatment temperatures and the content of N-doped carbon reveals that both these factors significantly affect ORR performance. Heat treatment improves the phase and crystallinity of the MnO nanoparticles, whereas the N-doped carbon content affects the MnO nanoparticle size and dispersion. The optimized MnO/N-CC-2-900-2 catalyst exhibited ORR activity identical to the commercial Pt/C catalyst in 0.1 M HClO_4_.

In another study from the same research group, the effect of the N-doped carbon content on ORR activity and MnO nanoparticle size and dispersion was further investigated [114]. It can clearly be seen that the N-doped carbon content has a significant effect on the MnO nanoparticle size and the nanoparticle distribution. During the gelation process, the N-doped carbon was trapped in the interconnected gel in situ, during the formation of the xerogel. The sequentially increased N-doped carbon content during gelation meant that more and more N-doped carbon was trapped inside the gel, helping to separate the MnO and thus improving the dispersion and distribution by mitigating the agglomeration of the nanoparticles during high-temperature treatment. The reduced particle size helps in enhancing the active surface area available during electrochemical reactions. The effects of nanoparticle size and distribution are clearly seen from the XRD analysis of the MnO/N-CC catalysts, where a significant reduction in the MnO nanoparticles’ size is observed with the increase in the N-doped carbon content. Similar observations were also drawn from the SEM and TEM measurements, where the MnO distribution improved when fewer and fewer agglomerations were seen with increased N-doped carbon content. The optimized MnO/N-CC-5 catalyst exhibited a half-wave potential of 0.78 V vs. RHE in the 0.1 M KOH electrolyte, slightly lower than that of the commercial Pt/C catalyst.

Among several non-precious metal catalysts, Fe-N-C catalysts in the form of atomically dispersed states are the best-known catalysts so far [115]. The highly electrocatalytically active ORR catalyst with dense Fe-N_4_-C active sites is generally synthesized via co-precipitation, wet impregnation, organic polymers, and most commonly via metal–organic framework precursors [116]. Nevertheless, the simple catalyst synthesis systems are deemed in view of commercialization, which requires cost effectiveness and simple synthesis steps. When obtaining the highly dense Fe-N_4_-C active sites via pyrolysis treatment of the precursors, inhomogeneous aggregation of single-atom Fe catalysts into clusters/agglomerations is commonly observed, which hinders the formation of Fe-N_x_ active sites, as well as reduces the density/atomic loading of the Fe on the carbon matrix [117,118]. In addition to the density of FeN_x_ active sites, the porosity of the catalyst is paramount. In general, micropore edges host the FeN_x_ active sites, whereas the meso-/macropores facilitate the diffusion of the reactants/ions to the active sites, and also help in the removal of the ORR products [119,120]. Therefore, it is crucial to develop robust and cost-effective methods to efficiently produce SACs with rational porosity and plenty of accessible FeN_x_ active sites for ORRs.

**Figure 7:**
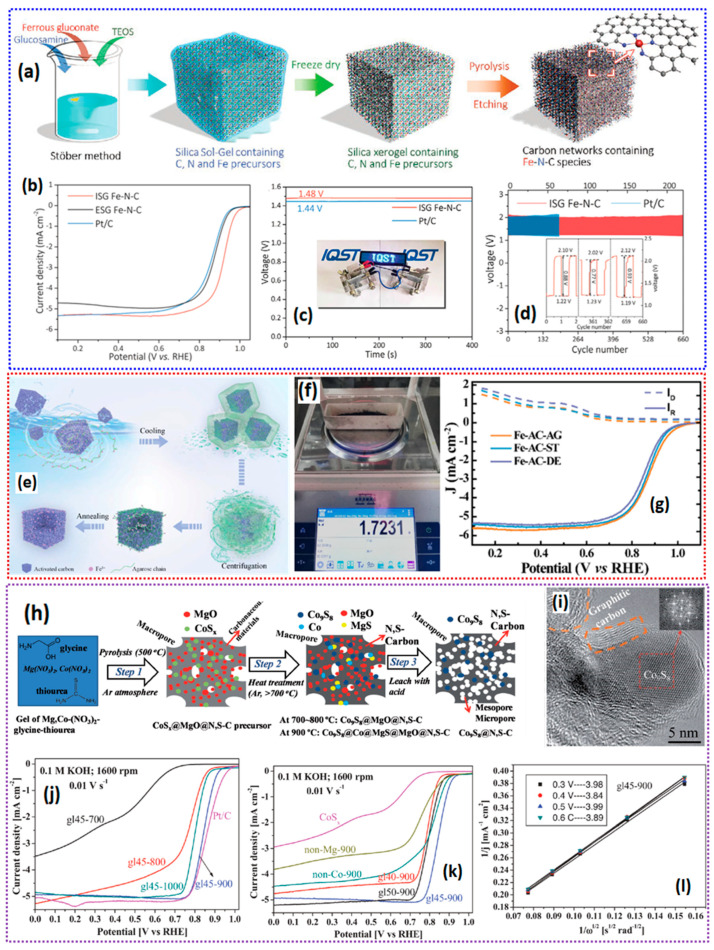
(**a**) Graphical illustration of the synthesis of single-atom ISG Fe-N-C catalyst. (**b**) LSV curves of ISG Fe-N-C, ESG Fe-N-C, and Pt/C catalyst. (**c**) OCV values of ISG Fe-N-C and Pt/C catalysts (inset: two Zn–air batteries connected to each other in series and illumination of the LED lights with Zn–air batteries), (**d**) discharge–charge curves of ISG Fe-N-C and Pt/C catalysts [Reproduced with permission from Ref. [121]], (**e**) graphical illustration, (**f**) gram-scale synthesis of Fe-N-C catalysts, (**g**) LSV curves of the Fe-N-C catalysts synthesized with polymers such as agarose (Ag), starch (ST), and dextrose (DE) [Reproduced with permission from Ref. [122]], (**h**) graphical illustration of Co_9_S_8_@NSC catalyst, (**i**) HR-TEM image of Co_9_S_8_@NSC catalyst (inset: SAED pattern), (**j**,**k**) LSV curves of various catalysts synthesized at different temperatures, and (**l**) K-l plots [Reproduced with permission from Ref. [123]].

In view of this, Liu et al. [121] proposed a novel synthesis approach in which ferrous gluconate and glucosamine precursors mediated an in situ silica xerogel (ISG) strategy to fabricate Fe-N-C SACs (Figure 7a). Most importantly, using this method, a scalable synthesis can be carried out with ~100 g of the catalyst per batch, with a final catalyst yield of ~41%. These highly reproducible Fe-N-C SACs can be economically important, not only because of the use of naturally occurring precursors, but also due to the high yield of the final product, which can be scaled up to the industrial level. The synthesis of the catalyst includes a simple three-step process. The first step included the hydrolysis of the TEOS in the presence of the ferrous gluconate and glucosamine precursors, leading to the homogenous incorporation of these precursors in the 3D SiO_2_ sol–gel. This was followed by solvent evaporation, yielding the SiO_2_ xerogel. In the second step, the obtained 3D SiO_2_ xerogel was then subjected to pyrolysis in an inert atmosphere, during which the precursors underwent carbonization, yielding the Fe-N-C catalyst trapped inside the 3D SiO_2_ network. In the final step, the hard template silica was removed using HF, which dissolves the Si framework that generates the Fe-N-C SAC. The HR-TEM measurements indicate that the ISG Fe-N-C SAC contains a highly porous 3D interconnected graphite-like carbon network with almost no visible aggregate of Fe, suggesting the atomically dispersed state of Fe in the catalyst. The ISG Fe-N-C SAC exhibited a high BET surface area of 704 m^2^ g^−1^. Such a high surface area possibly results from the opening of mesopores due to the removal of SiO_2_. The H_3_-hysteresis loop of the ISG Fe-N-C SAC signifies a type-II isotherm, confirming the presence of micro-/meso-/macropores in the catalyst. In RDE studies, the ISG Fe-N-C SAC, with an Fe loading of 1.94 weight percent (wt%), exhibited the highest half-wave potential of 0.98 V in 0.1 m KOH, and a half-wave potential of 0.74 V in 0.5 M H_2_SO_4_ solution (Figure 7b). In an effort to identify the active sites, the Fe-N_x_ poisoning sites were constructed, in which the ISG Fe-N-C SAC showed a considerable reduction in the charge associated with the site density, confirming the presence of accessible Fe-N_x_ moieties as active sites. In addition to the extraordinary ORR activity, the ISG Fe-N-C SAC also exhibited excellent stability, with a loss of just 8 mV after 5000 potential cycles. In a Zn–air battery setup, the ISG Fe-N-C SAC delivered a power density of 259 mw cm^−2^, much higher than that of the Pt/C catalyst (140 mw cm^−2^). The specific capacity of the ISG Fe-N-C SAC is also higher than that of the Pt/C catalyst, with 763 mAh g^−1^, excellent discharging capacity, and durability for 600 cycles (Figure 7c,d). This study suggests that the ISG Fe-N-C SAC could potentially be replaced with the commercial Pt/C catalyst due to the high ORR activity and stability, in addition to its potential to be scaled up to industrial levels.

To meet commercial requirements, a catalyst must exhibit high electrocatalytic activity and long-term durability. Additionally, in accordance with the industrial standards for mass production from the gram to kilogram levels, the synthesis procedure should be straightforward and scalable [124]. Because of their high surface energy, single-atom catalysts with higher atomic loading in particular have a tendency to form clusters and agglomerates during this process, which lowers the activity and effective electrochemical surface area [125]. Several mitigation strategies have been adopted to synthesize highly dispersed SACs, such as atomic layer deposition, MOF- and COF-derived catalysts via pyrolysis, and vapor atom trapping methods [126]. However, these methods come with their own disadvantages, such as the requirement of sophisticated instruments and the selective leaching of agglomerated particles via post-synthesis processes. Wang et al. [122] proposed a simple and feasible large-scale synthesis of single-atom Fe-N-C catalysts via a gel-limiting strategy for the ORR. The gel-limiting approach follows the simple electrostatic interaction of the hot agarose aqueous solution with the dissolved Fe^n+^ ions, followed by the addition of the activated carbon (Figure 7e). The aqueous agarose-Fe^n+^ gel diffuses into the pores of the activated carbon, which, upon cooling, undergoes solidification into a gel. The resulting gel is subjected to the pyrolysis process in NH_3_ to fix the N atoms and Fe-N_x_ moieties into the carbon matrix as Fe-N-C active sites [127]. The resulting Fe-AC-2 catalyst showed no visible agglomeration of the Fe particles, suggesting that all the Fe atoms are in an atomically dispersed state. The obtained Fe-AC-2 catalyst exhibited a BET surface area of 950 m^2^ g^−1^ and a pore volume of 0.77 cm^3^ g^−1^. This synthesis process is termed as a gel-limiting strategy due to the ability of the agarose gel to take up a limited amount of Fe. When the Fe wt% was increased from 1 to 46.3%, the actual iron content obtained after ICP analysis was found to be 0.71–6.5%. This indicates that despite the high concentration of Fe ions available, there is a limit to the Fe ions’ uptake by the agarose gel. The rest of the unfixed Fe ions are removed during the centrifugation process of the gel, before pyrolysis. One of the important aspects of this study is the ability to produce gram-level catalysts per batch, namely 1.7231 g/batch, indicating that this method has the potential to be scaled up to the kilogram level in industrial processes (Figure 7f). The Fe-AC-2 catalyst delivered an onset and half-wave potential of 1.00 V and 0.87 V vs. RHE in 0.1 M KOH electrolytes, respectively, slightly higher than the values of the commercial Pt/C catalyst. In the Zn–air battery setup, the Fe-AC-2 catalyst delivered a power density of 153 mW cm^−2^, with excellent cyclic stability. In order to create the Fe-N-C catalyst, the authors also extended the synthesis process to other hydroxyl-bearing polymers, such as starch (ST) and dextran (DE), in addition to agarose (AG) (Figure 7g). Surprisingly, the ORR activity of other polymer-derived catalysts was also found to be comparable to that of the agarose gel strategy, demonstrating the synthesis process’s adaptability.

Another scalable synthesis of the non-precious metal sulfide supported on N-S-doped carbon (Co_9_S_8_@NSC) synthesized via a famous solution combustion synthesis (SCS) of nitrate–glycine gel was carried out by Zhu et al. [123]. The catalyst was obtained by pyrolyzing and annealing a gel mixture of Mg, Co, and nitrate–glycine–thiourea under an Ar atmosphere, with subsequent HCl acid washing (Figure 7h). The simple and scalable synthesis of Co_9_S_8_@NSC is carried out via a self-sustaining exothermic redox reaction via an auto-combustion SCS process [128,129]. The synthesis process starts with the formation of a dried gel from the combustion reaction between glycine and metal nitrates of Mg and Co in the presence of thiourea at an ignition temperature of 300 °C [130]. The ignition is accompanied by the release of a large amount of combustible gases that create mesopores, in addition to the pores induced by the removal of the MgO template via acid washing. The resulting ignited powers then undergo heat treatment at temperatures above >700 °C to finally obtain the Co_9_S_8_@NSC catalyst. The XRD analysis reveals the crystalline nanoparticles of Co_9_S_8_, and the TEM measurements show the graphitic carbon of 3D graphene or graphene-like architectures and the crystalline nanoparticles of Co_9_S_8_, with a 3D porous structure (Figure 7i). The crystalline graphitic carbon is assumed to be formed due to high-temperature catalytic graphitization with metallic cobalt. The catalyst with different amounts of carbon in relation to Co_9_S_8_ can be obtained simply by changing the amount of glycine precursor. This claim is validated by synthesizing the catalysts with different ratios of glycine, and the catalysts with different carbon contents were obtained, which were calculated from the TGA analysis. The gl45-900 catalyst, with a carbon content of 60%, showed a BET surface area of 409 m^2^ g^−1^ and a wide range of porosity, with micro-, meso-, and macropores. The gl45-900 catalyst delivered excellent ORR activity, with an onset potential of 0.96 V and a half-wave potential of 0.85 V, almost equal to those of the commercial Pt/C catalyst, and a dominant direct four-electron reduction of O_2_ (Figure 7j–l). It was also observed that the pyrolysis temperature and the ratio of glycine to nitrate precursor strongly influence the ORR activity of Co_9_S_8_@NSC catalysts. It is obvious that the pyrolysis temperatures affect the catalyst’s morphology, size, crystallinity, and distribution of the Co_9_S_8_ nanoparticles, and the graphitic nature of carbon, in addition to its role in the decomposition of the sulfur in the catalyst at higher temperatures. On the other hand, the ratio of glycine–metal nitrites affects the amount of carbon that is derived from the glycine precursor and the loading of the metal nanoparticles. The excellent activity of the Co_9_S_8_@NSC catalyst was attributed to the crystalline Co_9_S_8_ particles and high electronic conductivity of the 3D graphitic-like carbon sheets induced by N and S dopants [131]. In conclusion, the work presented here represents a simple and novel method for creating N- and S-doped graphene-like porous carbon embedded with Co_9_S_8_ in one pot for hybrid ORR electrocatalysts. Only low-cost, non-toxic industry chemicals and simple equipment were used in the experiment, enabling scalable preparation. The proposed strategy offers a cost-effective and efficient method for creating hybrid composites with a rational structural design, suitable for producing graphene-like carbon-based materials.

## 6. Metal–Organic Gel (MOG)- and Supramolecular Gel-Derived Catalysts

The design and synthesis of one-dimensional nanostructured materials via self-assembly has attracted tremendous interest in recent years. The self-assembly of low-molecular-weight gels composed of large amounts of solvents, a solid gelator, and solvent molecules is immobilized within the gel matrix formed by the hierarchical self-assembly of gelator molecules. When a pot of gel is turned upside down, it stays stable under the force of gravity, making it easy to identify using a straightforward “inversion test” [132]. During the gelation process, if metals or metal complexes are present either in the solid phase as a gelator or the liquid phase, metal-containing gels are formed. MOGs, which are solid-like materials with viscoelastic properties, are typically formed through the combination of metal–organic coordination forces with other interactions, such as π−π and H-bonding, and can also extend to three-dimensional interconnected structures [133]. MOGs typically exhibit a high mass flux, thermal stability, and adjustable pore structures; they are also formed quickly under mild circumstances, with the raw materials completely utilized. One distinguishing characteristic of MOGs that sets them apart from solids is the huge quantity of liquid (solvent) contained in their sponge-like gel matrices. In this manner, MOGs can serve as a rich pool where tailored materials can be embedded and distributed across the entire gel matrix, leading to sufficient interaction among all the species that make up MOGs. Accordingly, MOGs are ideal precursors for the production of nano-sized ORR electrocatalysts due to their excellent inclusion properties, design flexibility, and ease of synthesis. MOG-derived catalysts find several applications in material templates, gas separation, catalysis support, and dye removal applications [134]. However, little attention has been paid to their application in electrocatalysis.

The metal-containing gels are classified as “metal–organic gels (MOGs)” or “supramolecular gels” based on the nature of the interaction between them. MOGs are formed between metallic ions and organic ligands, via a strong covalent bond or metal–ligand coordination [135], whereas supramolecular gels are formed via the self-assembly of low-molecular-weight organic ligands or polymers via weak π−π interactions, hydrogen bonding, and hydrophobic interactions between them [136]. The MOGs and “supramolecular gels” are subjected to pyrolysis, either alone or in combination with other 2D and 3D structures such as graphene/MOF, resulting in carbon-supported metals that can be used as electrocatalysts in various energy storage and conversion applications [137]. Not many studies are devoted to this area, especially for the electrocatalysis of the ORR. The following section discusses recent studies that represent electrocatalyst synthesis via MOGs and supramolecular gels for the ORR.

### 6.1. Covalent/Coordination Boned MOG-Derived Catalysts

**Figure 8:**
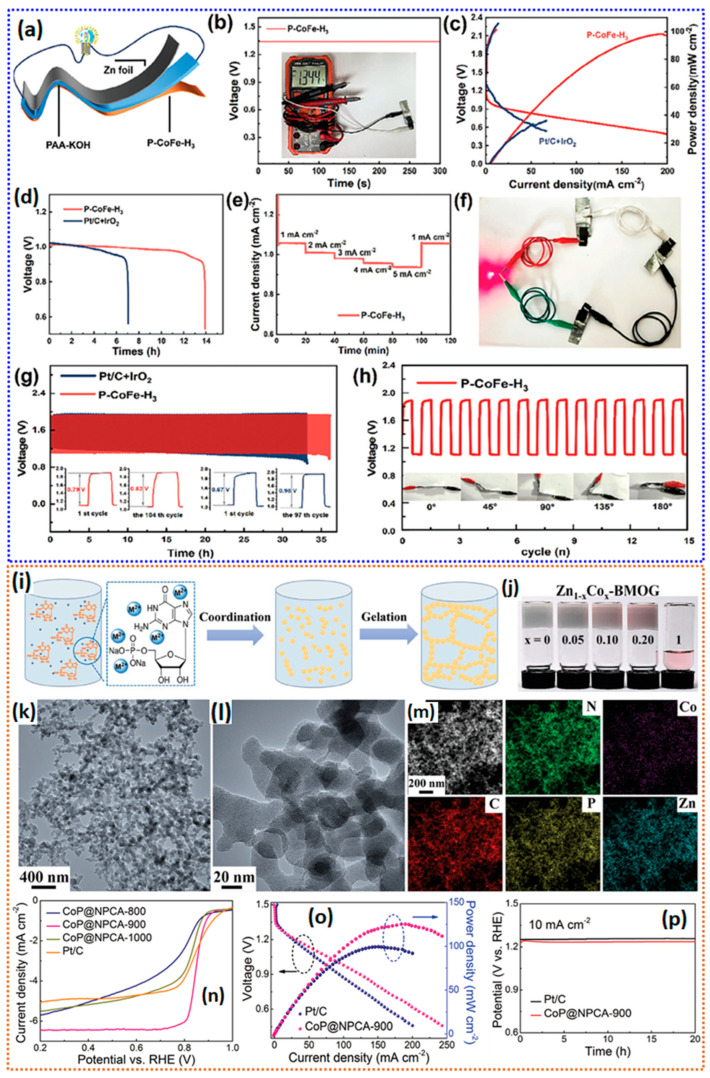
(**a**) Graphical illustration of solid-state flexible zinc–air battery. (**b**) OCV values of P-CoFe-H3 as cathode catalyst (inset: visual representation of OCV values), (**c**) discharge/charge curves of P-CoFe-H3 and Pt/C+IrO_2_ catalysts, (**d**) discharge curves of the solid-state Zn–air battery at 2 mA cm^−2^, (**e**) galvanostatic discharge curves at various current densities, (**f**) practical demonstration of the P-CoFe-H3 catalyst by illuminating the LED by connecting a series of Zn–air batteries, (**g**,**h**) long-term galvanostatic discharge curves at 2 mA cm^−2^ (inset to figure h: applying bending strain at different angles in every 3 cycles) [Reproduced with permission from Ref. [139]]. (**i**) Graphical illustration of the synthesis of the Zn1-xCox-BMOG catalysts and (**j**) their visual representations in inverting positions. (**k**,**l**) TEM images and their (**m**) corresponding HAADF-STEM images of Zn0.90Co0.10-BMOG catalysts with their elemental mapping of N, C, P, Co, and Zn. (**n**) ORR polarization curves, (**o**) Zn–air battery discharge curves, and (**p**) OCV values of CoP@NPCA-900 and Pt/C catalysts [Reproduced with permission from Ref. [141]].

With their 3D cross-linked architecture, diverse ligands, and distinct metal centers, metal–organic gels are strong coordination bond-mediated gels between organic ligands and metal ions [135]. MOGs possess a 3D cross-linked architecture, diversified ligands, and a definite metal center. Several MOG-derived catalysts have been synthesized using organic ligands such as 4,4′,4″tricarboxyphenylamine, phytic acid, and tricarboxylic acid in combination with Fe^3+^, Co^2+^, and Ni^2+^ ions, and the catalysts derived from these gels have been utilized for various energy-related applications, such as OERs and HERs [138]. Furthermore, the use of multidentate organic ligands or multiple ligands could diversify the synthesis process, which could form several topological MOGs, such as with chains, sheets, interpenetrating networks, and polyhedral shapes. Using more than one ligand would increase the possibility of coordination bonds with metallic ions, and hence enhance the possibilities of forming high-density active sites. In view of this, Dong et al. [139] proposed a pyrolysis-free MOG-based electrocatalyst via the dual-ligand approach to synthesize FeCo MOGs with phytic acid and tricarboxylic acid ligands (H_3_TATAB). The synthesis of P-CoFe-H_3_ includes the initial metal coordination bonds between phytic acid and CoFe ions that form P-CeFe with unsaturated coordination metal sites and an O-coordination environment, with the addition of a second ligand that coordinates with P-CeFe via -N coordination for the metal ions in the solution, which results in the formation of a gel. The XRD analysis of the P-CoFe-H_3_ shows the amorphous structure that is expected, because the P-CoFe-H_3_ is not subjected to pyrolysis. However, the P-CoFe-H_3_ catalyst exhibited trifunctional activity for electrochemical ORRs, OERs, and HERs. The P-CoFe-H_3_ catalyst delivered an ORR half-wave potential of 0.80 V, a HER overpotential of 260 mV at 10 mA cm^−2^, and an OER overpotential of 257 and 276 at 10 and 50 mA cm^−2^, respectively. The bifunctional ORR and OER potential gap was found to be 0.69 V smaller than that of the IrO_2_/Pt/C catalyst. The practical application of P-CoFe-H_3_ catalysts is demonstrated in flexible Zn–air batteries, in which the P-CoFe-H_3_ as a cathode delivered a power density of 98 mW cm^−2^, whereas the IrO_2_/Pt/C catalyst could only deliver 36 mW cm^−2^ (Figure 8a–c). In addition, the higher-power-density CoFe-H_3_ catalyst also exhibited good cycling stability under different folding angles, suggesting the robust nature of the CoFe-H_3_ catalyst, suitable for flexible electronic applications (Figure 8d–h). Wang et al. [140] fabricated pyridine-Fe gel-derived ultra-low loading of the Pt catalyst for the ORR. The pyridine-Fe gel was first obtained using 2-aminopyridine as a ligand and Fe (NO_3_)_2_ as a gelator, and the resulting gel was immersed in an aqueous solution of H_2_PtCl_6_, during which the Pt ions coordinated with the unsaturated pyridine-Fe gel, followed by pyrolysis, to yield the N_3_/Fe/C-Pt catalyst. In phosphate buffer solution, there was an electrolyte positive onset potential of 0.19 V (vs. Ag/AgCl) and a half-wave potential of 0.03 V (vs. Ag/AgCl), which is comparable to the results with commercial Pt/C catalysts.

MOGs are a class of materials characterized by metal–ligand bonds that can form conductive, high-surface-area composite materials upon pyrolysis. MOGs can be synthesized easily in large quantities under simpler and milder conditions than the related MOFs. Seldom, MOG-derived catalysts show agglomerated nanoparticles due to their structural instability at higher temperatures and rapid decomposition of the organic ligand. Furthermore, MOG-derived catalysts lack sufficient porosity, due to the lack of structural directing agents or porogens in the precursors, unlike MOFs. Therefore, it is highly challenging to obtain MOG-derived catalysts with good dispersity of the metallic active sites with a hierarchical porous structure. In view of this, Guo et al. [141] proposed a self-templated carbonization strategy to synthesize heterostructured CoP@NPCA catalysts via a metallogel containing bimetallic clusters (BMOG) as the precursor (Figure 8i–j). Initially, the aqueous solution of guanosine monophosphate (GMP) is mixed with an aqueous solution of Zn and Co ionic salts that results in a gel that is pyrolyzed to obtain the CoP@NPCA catalyst. Rich in C and N content, the GMP provides sufficient accessible coordination sites for the metallic ions. Thanks to the coordination environment between GMP and Zn/Co ions, the morphological study reveals that the CoP@NPCA catalyst preserves a structure quite similar to that of its precursor. The catalyst shows a three-dimensional quasi-aerogel configuration. This structure results from a simple mixing of GMP with the metal precursors, which generates a layered morphology. In particular, the stabilization and preservation of the quasi-aerogel framework over the pyrolysis process depend critically on the coordination bonds between GMP and metal ions. The versatility of the synthesis process is established by replacing the Co atoms with other transition metal ions such as Ni and Fe ions, which also results in a similar morphology and electrocatalytic activity (Figure 8k–m). Alkaline environment CoP@NPCA catalysts, FeP@NPCA catalysts, and NiP@NPCA catalysts exhibited an ORR half-wave potential of 0.85, 0.83, and 0.83 V vs. RHE, respectively. In a zin-air battery setup, the CoP@NPCA catalyst delivered a power density of 125 mW cm^−2^ and a higher voltage at a constant current of 10 mA cm^−2^ than the commercial Pt/C catalyst, and high gravimetric energy densities of 835.4 and 745.2 W h kg^−1^, respectively, at 10 and 50 mA cm^−2^ current densities (Figure 8n–p).

**Figure 9:**
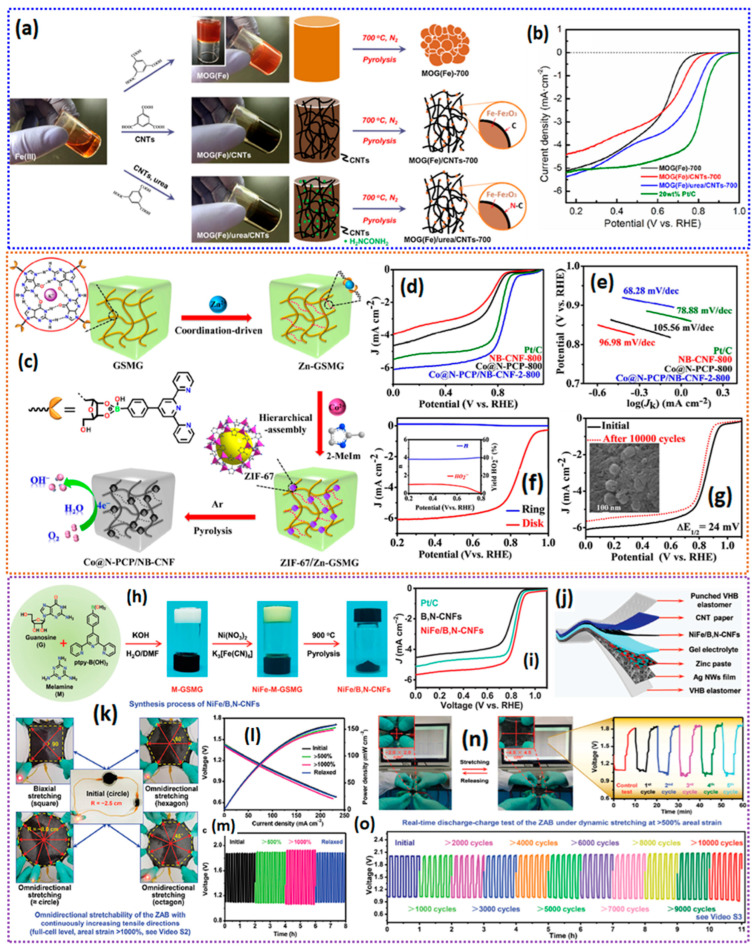
(**a**) Schematic representation of Fe(MOG)-based catalysts and (**b**) their ORR polarization curves [Reproduced with permission from Ref. [143]]. (**c**) Graphical representation of Co@N-PCP/NB-CNF catalysts, (**d**) ORR polarization curves, (**e**) Tafel of Pt/C, NB-CNF-800, Co@N-PCP-800, and Co@N-PCP/NB-CNF-2-800 catalysts, (**f**) LSV curves of the ring and disk currents (inset: peroxide and number of electrons), (**g**) LSV curves of Co@N-PCP/NB-CNF-2-800 before and after 1000 cycles (inset: SEM morphology image of the catalysts after 10,000 cycles) [Reproduced with permission from Ref. [144]]. (**h**) Schematic representation of (**i**) ORR curves of NiFe/B,N-CNFs catalysts, (**j**) graphical representation of the flexible Zn–air battery, (**k**) pictures, (**l**) performance of the flexible Zn–air battery at various areal strains, (**m**) cycling profiles, (**n**) photographs of the experimental site and real-time ZAB discharge and charge test curves during repeated dynamic stretching–releasing at room temperature, and (**o**) ZAB cycling stability during 10,000 stretching cycles at 400% aerial strain [Reproduced with permission from Ref. [145], open access].

By combining an organic ligand with an inorganic catalyst synthesis based on hybrid interpenetrating networks, MOGs make it possible to create new materials with controllable characteristics, like porosity, which is mainly due to their large interspace free volume. Shijina et al. [142] reported a hybrid organic–inorganic-based polymer network that forms a stable hybrid MOG, which upon pyrolysis generates highly porous graphitic sheets with firmly anchored Fe and N. The melamine–formaldehyde (MF) polymer serves as an inorganic component, and Fe- benzene-1,3,5-tricarboxylic acid (H_3_BTC or trimesic acid) serves as an organic MOG. The rheological analysis of the hybrid interpenetrating network using an amplitude sweep test reveals the cross-over strain value of 5.95% obtained for the inorganic-Fe-MOG hybrid, indicating the better stability of the formed gel network. The aim of the study is to enhance the surface area of Fe-MOG-MFN via a naphthalene, a sublimable porogen, to induce mesoporosity. The morphological analysis of the catalysts with and without naphthalene exhibited contrasting microstructures. With naphthalene as the porogen, the catalyst exhibited sheet-like graphitized carbon and highly interconnected macropores, whereas the catalyst without naphthalene showed fewer mesopores in the catalyst. The addition of the naphthalene as a porogen helped in the generation of additional carbon in the catalyst, which resulted in a higher surface area of 950 m^2^ g^−1^ and enhanced mesoporosity. Despite the enhanced surface area, the catalyst showed only moderate ORR activity, implying that further improvements were required to enhance the microstructure of the catalyst. The Fe-MOG-MFN-C catalyst exhibited an ORR onset potential of 0.91 V, much less than that of the Pt/C, which was 1.0 V vs. RHE. In addition, the Fe-MOG-MFN-C catalyst also exhibited a higher HO_2_^−^ yield of about 20%, and the number of electrons transferred per O_2_ molecule was calculated to be 3.6, which is not a desirable factor for fuel cell or Zn–air battery applications. Except for a few, most MOG-derived catalysts have shown just acceptable ORR activity, mainly due to large agglomerated metallic nanoparticles resulting from pyrolysis. One of the studies aimed at reducing the particles’ size by combining the MOGs with CNTs, as proposed by Wang et al. [143]. A mixture of 1,3,5-bezenetricarboxylic acid with Fe ionic solution resulted in a brown gel that underwent the pyrolysis process, showing highly agglomerated Fe particles with a particle size ranging from 10 to 150 nm. However, with the addition of CNTs and urea, the gelation process resulted in a black-colored gel. The CNT-incorporated gel-derived MOF(Fe)/urea/CNTs-700 catalyst contrastingly showed the homogenous distribution of the Fe nanoparticles of a size of around 10 nm, implying that the presence of CNTs tunes the catalyst morphology by reducing the Fe nanoparticle size and thus enhancing the ORR activity compared to that of their control catalysts. Despite all the efforts, the resulting catalyst still exhibited inferior ORR activity in relation to the Pt/C catalyst, requiring further studies to improve the catalyst structure and ORR activity (Figure 9a,b).

### 6.2. Supramolecular MOG (SMG)-Derived Catalysts

Because of their porous network, high compositional tunability, ease of synthesis and functionalization, and abundance of defects for mass transfer, supramolecular gels (SMGs) assembled from small molecules via various noncovalent interactions are especially appealing for catalytic applications [146]. The electrocatalytic applications of catalysts derived from SMGs are limited, however, by their poor crystallinity. Gu et al. [144] proposed SMG-ZIF-67 hybrid structures to improve the crystallinity of the SMG-derived catalysts based on guanosine supramolecular gel (GSMG) as the host material to ZIF-67 (Figure 9c). The GSMG-derived nanofiber surface is modified with ptpy-B(OH)_2_ ligand, which helps to bind ZIF-67 structures via N groups of pyridine moieties. The Co@N-PCP/ NB-CNF-800 catalyst retained the fiber network structure and the polyhedral shapes of the parent materials, with homogeneous ZIF-67 on the GSMG nanofibers, along with the formation of graphitized carbon, helping to enhance the crystallinity and electronic conductivity of the catalysts. XRD analysis revealed the presence of metallic Co nanoparticles, and XPS analysis showed metallic Co and Co-N_x_ sites as possible ORR active sites in the catalysts. The Co@N-PCP/NB-CNF-800 catalyst exhibited an ORR onset potential of 1.01 V vs. RHE and a half-wave potential of 0.85 V vs. RHE, which are higher than for the commercial Pt/C standard. The Co@N-PCP/NB-CNF-800 catalyst also showed the lowest Tafel slope of 68 mV dec^−1^, with a nearly four-electron transfer. In addition, the Co@N-PCP/NB-CNF-800 catalyst also exhibited excellent stability, with a loss of just 24 mV after 10,000 potential cycles. In a zinc–air setup, the Co@N-PCP/NB-CNF-800 catalyst delivered a power density of 143 mW cm^−2^, much higher than that of the commercial Pt/C catalyst, with good cyclic stability (Figure 9d–g).

Because of their compositional tunability, ease of synthesis, and hierarchical porous 3D network, and the recent interest in their potential role in stretchable/flexible energy storage applications, supramolecular gels have garnered a lot of attention. Nevertheless, additional research into supramolecular gels for incorporation in commercial devices is necessary due to their poor crystallinity and structural instability at high temperatures. In view of this, Liu et al. [145] proposed an excellent strategy for a highly crystalline and highly efficient NiFe/B,N-CNF catalyst via a guanosine-based supramolecular hydrogel (GSMG) route (Figure 9h), based on their earlier study [144]. The melamine was introduced into the GSMG structure, along with the Ni^2+^ and [Fe(CN)_6_]^3−^, to coordinate the terpyridine groups on the fiber surface to further enhance the structural stability of M-GSMG. The pyrolyzed catalyst NiFe/B,N-CNFs showed the lattice planes corresponding to the NiFe alloy.

The HRTEM analysis also showed that some NiFe alloy nanoparticles are encapsulated by graphite carbon, thus exhibiting core/shell-like nanoparticles. The NiFe/B,N-CNF catalyst in the 0.1 M KOH electrolyte exhibited onset and half-wave potentials of 0.94 and 0.84 V, respectively, which are slightly higher than those of the corresponding Pt/C catalysts, which are 0.91 and 0.82 V vs. RHE, respectively (Figure 9i). The full cell-level aerial strain was more than 1000% when the circular omnidirectional stretchable ZAB (with a radius of 2.5 cm) was stretched to 8.0 cm. The ZAB demonstrated exceptional cycling stability (>130 h), a high power density of 159.0 mW cm^−2^, and a steady open-circuit voltage of approximately 1.47 V. It also exhibited great rate discharge characteristics. Even after being stretched to areal strains of 500% and 1000%, the ZAB maintained power densities of 153.0 and 148.8 mW cm^−2^, respectively. The constructed stretchable ZAB also showed excellent flexibility, even after repeated stretches under stress-release experiments (10,000 stretching–releasing cycles at ~400%), unveiling its great potential for commercial applications (Figure 9j–o). Despite being dynamically stretched and released at an aerial strain of 500%, the ZAB was able to charge and discharge steadily within the cm voltage range of 1.00–1.86 V at 2 mA^−2^, indicating that it could consistently output power upon deformation.

**Figure 10:**
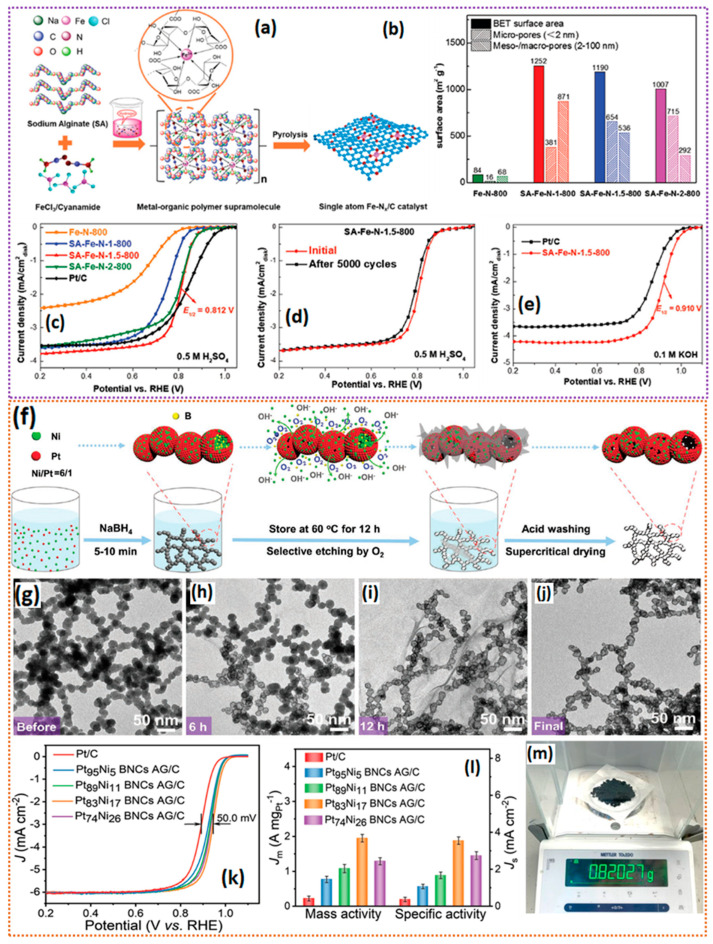
(**a**) Graphical representation of the single-atom Fe-N-C catalyst’s synthesis, (**b**) histograms representing the BET surface area and porosity of the various Fe-N-C catalysts, (**c**) ORR polarization curves of various Fe-N-C catalysts and Pt/C catalysts, (**d**) stability curves of SA-Fe-N-1,5-800 catalyst, and (**e**) LSV comparison curves of Pt/C and SA-Fe-N-1,5-800 catalysts in 0.1 M KOH electrolyte [Reproduced with permission from Ref. [150]]. (**f**) Graphical representation of the formation mechanism of Pt_83_Ni_17_ BNCs AG and (**g**–**k**) TEM images of the Pt-Ni recorded at various time intervals. LSV polarization curves (**l**), mass and kinetic currents of various Pt_x_Ni_y_ BNCs AG catalysts, and (**m**) gram-scale synthesis of the Pt_83_Ni_17_ BNCs AG catalysts [Reproduced with permission from Ref. [153]].

Generally, highly active M-N_x_-C catalysts are prepared via a pyrolyzing mixture of metal salts, carbon precursors, and nitrogen sources [147]. Despite this process appearing to be easy, the resulting catalyst always seems to have agglomerated nanoparticles that hinder the maximum utilization of the metallic active sites [148]. Furthermore, the metallic aggregates or nanoparticles tend to dissolve in the acidic or alkaline electrolytes, leading to reductions in the ORR activity [149]. For the maximum utilization of the metallic active sites, atomically dispersed metallic sites are important, which is challenging, owing to the high surface energy of single-atom catalysts, which tend to form clusters/aggregates. In view of this, Miao et al. [150] proposed a self-locking supramolecular synthesis strategy by using sodium alginate, which possesses excellent hydrophilic functionalities. The special property of SA is that it spontaneously bonds with Fe atoms via chelation [151]. Upon mixing the aqueous Fe^3+^ ions with SA under thermal heating at 60 °C, a red gel was formed. After drying, pyrolysis, acid leaching, and secondary heat treatment, an atomically dispersed Fe atomic catalyst, SA-Fe-N-1.5-800, was obtained (Figure 10a). This metal–organic polymer supramolecular structure, commonly referred to as the “egg-box” model, is what actually forms during alginate gelation when Fe^3+^ chelates with oxygen-containing groups of G-blocks in SA [152]. The SA and Fe^3+^ ions form cross-linking supramolecular structures with hydroxyl/carboxyl groups of SA, forming a 3D SA-Fe hydrogel framework. The 3D SA-Fe hydrogel framework is formed in cyanamide, an inorganic N source. Morphological analysis of the SA-Fe-N-1.5-800 catalyst reveals a highly porous architecture, with no visible agglomeration of Fe particles or carbon nanotube structures formed during pyrolysis, suggesting that Fe atoms exist only in the form of an atomically dispersed state. The SA-Fe-N-1.5-800 catalyst also exhibited the highest BET surface area of 1190 m^2^ g^−1^, one of the highest in supramolecular gel catalysts (Figure 10b). In a 0.5 M H2SO4 aqueous solution, SA-Fe-N-1.5-800 delivered a half-wave potential of 0.812 V vs. RHE, slightly lower than that of the commercial Pt/C catalyst, an admirable stability of 0.5 M H_2_SO_4_ (Figure 10c–e), and excellent stability, with almost no loss in the half-wave potential after 5000 cycles. The ORR activity was found to be significantly improved after acid leaching, which helps in eliminating the amorphous and dissolvable Fe-oxide species from the catalyst. The non-acid-leached catalyst showed lower ORR activity than the acid-leached catalyst, indicating that acid leaching is an essential step for enhancing ORR activity. The effect of acid leaching was also seen in the number of electrons transferred per O_2_ molecule, where the non-acid-leached catalyst showed a maximum ‘n’ of 3.45, whereas the acid-leached catalyst’s ‘n’ was close to 4.0. In the 0.1 M KOH electrolyte, the SA-Fe-N-1.5-800 catalyst outperformed Pt/C in ORR activity, surpassing Pt/C with a half-wave potential of 0.910 V vs. RHE.

## 7. Metal Aerogel-Derived Catalysts

The heterogeneous electrocatalytic reaction kinetics primarily depend on an accessible electrochemical surface area, high electronic conductivity, and the mass transfer of the reactants to the metallic active sites. Metal aerogels (MAs) are a new class of metallic porous materials that started to attract special attention in 2009 [154,155]. Metal gels or metal aerogels are entirely composed of metallic atoms that inherit all the intrinsic properties of the metals they are composed of, in addition to the porous network [156,157]. Metal aerogels can also be termed as support-free catalysts, in contrast to traditional catalysts, in which nanoparticles are deposited on high-surface-area carbon supports. In this context, with multiple active sites, swift mass/electron transfer pathways, a resilient structure, and adjustable compositions, MAs exhibit exceptional potential as highly active and durable electrocatalysts, surpassing traditional non-metal and unsupported metallic catalysts [158]. The simple and straightforward method of using metal aerogel catalysts follows a simple reduction of metallic ions in an aqueous solution to form sol, followed by wet gel formation, which is basically the same as the sol–gel method, followed by a drying process, either supercritical drying or freeze-drying [159]. MAs have been explored as promising electrocatalysts for fuel cells due to their admirable activity and durability. Their high conductivity, porosity, and interconnected open channels provide the mass diffusion of the reactants and rapid electron transfer to all metallic active sites for an enhanced ORR. The modification of metal aerogel backbone structures to enhance their electrocatalytic performance has garnered significant interest. Nonetheless, this presents difficulties, due to the immense three-dimensional and inherently porous network architecture of metal aerogels. One of the most important aspects of MAs is the difficulty in their scalability, which remains a challenging task.

Zheng et al. [153] reported a scalable and precise controllable synthesis of Pt-Ni nanocage-structured aerogel catalysts (Pt_85_Ni_17_ BNCs AG) for the ORR. The aerogel catalyst was prepared via a simple reduction of aqueous solution containing Pt and Ni ions, reduced using a NaBH_4_ aqueous solution, resulting in a black-colored solution indicating the reduction of metals, which forms Pt-Ni hydrogel blocks dried via supercritical CO_2_ drying (Figure 10f). The morphological analysis of the Pt_85_Ni_17_ BNCs AG catalyst contains a 3D hierarchical interconnected porous network in the form of nanocages, with a wall thickness of 2.1 nm that corresponds approximately to 10 atomic Pt layers. Furthermore, these BNCs possess numerous grain boundaries, which are thought to enhance electrocatalytic performance [160]. The resulting MA in an x-ray diffraction analysis shows a crystalline, face-centered cubic crystal arrangement of PtNi alloy nanoparticles. The XPS analysis reveals a shift in the binding energy of PtNi alloys, attributed to the charge transfer from neighboring Ni to Pt, which lowers the d-band center, favoring the adsorption of O_2_ [161,162]. The time-dependent experimental observations indicate that, following the introduction of the reducing agent, the Pt-Ni hydrogel displays a necklace-like network structure formed by the random interconnection and fusion of solid nanospheres. The mean diameter of these nanospheres is (19.5 ± 2.8) nm, significantly larger than that of the nanocages in the Pt_83_Ni_17_ BNCs AG. The structural transformation of the Pt-Ni hydrogel was observed during the drying process. At 60 °C for 2 h, the Pt-Ni hydrogel exhibited solid nanospheres in hollow cages due to the etching of Ni species in the core. With the sturdy increase in time, an increasing number of nanocages were observed. With the initial Pt-Ni alloy solid structures, nanocage structures emerged due to the selective etching of Ni in the core. The dissolved oxygen was found to play a key role in the etching of core Ni. Due to the relatively inert nature of Pt, the core Ni reacts with the O_2_ and diffuses from the core to the surface via the Kirkendall effect [163,164]. This hypothesis was confirmed when the oxygen dissolved in the reaction solution bubbled out via N_2_, and the nanocage structures were not formed (Figure 10g–k). The Pt_83_Ni_17_ BNCs AG catalyst in the 0.1 M HClO_4_ electrolyte exhibited remarkable ORR activity, with a half-wave potential of 0.94 V vs. RHE, much higher than that of the commercial Pt/C catalyst, with a half-wave potential of 0.89 V vs. RHE. Interestingly, the mass activity and specific activity of the Pt_83_Ni_17_ BNCs AG catalyst at 0.9 V vs. RHE surpasses the DoE’s 2025 target, set by the U.S. Department of Energy, which is 0.44 A mgPt^−1^. The obtained specific and mass activities of the Pt_83_Ni_17_ BNCs AG catalyst are 3.55 mA cm^−2^ and 1.95 A mg mgPt^−1^, which is approximately 4.4 times higher than the DoE’s 2025 target (Figure 10l,m). In addition, the Pt_83_Ni_17_ BNCs AG catalyst exhibited extraordinary stability, with a loss of just 6.1 mV after 20,000 cycles, which is marvelous activity. The authors notably expanded the synthesis of the Pt_83_Ni_17_ BNCs AG catalyst using a scalable method, achieving gram-scale production by merely augmenting the reaction volume and precursor concentrations (Figure 10m).

In another study, Wu et al. [165] synthesized a hierarchically porous PdCuFe trimetallic alloy aerogel for an efficient ORR via the simple self-assembly of metallic ions by using a glycolic acid monohydrate (GAM) as the mild reducing and gelating agent, followed by lyophilization. With the addition of GAM, the metallic ions reduce to form a monolithic wet gel of a black color that settles at the bottom of the reacting vessel. The aging of the gel for 3 h at 50 °C resulted in a lightweight, 3D porous black powder. The XRD analysis shows the crystalline nature of a Pd_3_CuFe_0.5_ alloy of 5.6 nm with no other impurity phases. The Pd_3_CuFe_0.5_ catalyst exhibited a BET surface area of 75 m^2^ g^−1^ and a pore diameter of 15.29 nm. The SEM images show a highly micro- and mesoporous network, and the TEM measurements show a particle size of 5–10 nm. The ORR activity of Pd_3_CuFe_0.5_ catalysts in acidic, alkaline, and neutral pH electrolytes exhibited half-wave potentials of 0.92, 0.86, and 0.68 V vs. RHE, respectively. Interestingly, the Pd_3_CuFe_0.5_ catalyst produced a low amount of H_2_O_2_ of about 0.8–1.2%. In addition, in an aqueous Zn–air battery, an OCV of 1.44 V was displayed and a power density of 93 mW cm^−2^ was obtained. In a stability test conducted via the repeated charge/discharge of ZAB and at a current density of 10 mA cm^−2^ for about 120 h, almost no degradation in the voltage was shown, suggesting that aerogel electrocatalysts display an excellent charging/discharging cycling capability. In another study, Chen et al. [166] synthesized a Pt-Cu aerogel via a NaBH_4_ reduction method, which also serves as a stabilizer to the Pt-Cu aerogel. The PtCu-reduced products slowly settled down over a period of 4 h to obtain the black powder, which was collected via centrifugation, followed by being freeze-dried to obtain the final FeCu aerogel. The PtCu aerogel formation followed, via the simple addition of NaBH_4_ to the aqueous solution containing metallic ions, which underwent a rapid reduction to form the nuclei of the PtCu alloy. Simultaneously, the reduction side product BH_4_^−^ underwent decomposition to release H_2_ gas, which destabilized the PtCu hydrogel and sped up the coalescence of PtCu nanospheres via fusing and interconnecting, resulting in the formation of a PtCu alloy. The obtained PtCu aerogel looks like a sponge with interconnected hierarchical porosity. In an acidic electrolyte of 0.1 M HClO_4_ and at a scan rate of 5 mV s^−1^, the PtCu aerogel demonstrated a half-wave potential of 0.926 V vs. RHE, and excellent mass and specific activities. In an accelerated durability test, the PtCu aerogel catalyst delivered admirable stability, with a loss of just 20 mV for 5000 potential cycles, suggesting its excellent stability. A recent study by Wu et al. [167] demonstrated a cost-effective strategy for Pd_3_Cu derived from a mild reducing agent through self-assembly and freeze-drying techniques. The resultant catalyst exhibits a characteristic three-dimensional and “pearl-like” aerogel architecture. The Pd_3_Cu aerogel, through its structural characteristics and optimized chemical compositions, demonstrates exceptional pH-independent performance compared to the commercial Pt/C electrocatalyst. The optimized Pd_3_Cu aerogel exhibits exceptional ORR activity, achieving a half-wave potential of 0.90 V vs. RHE and a limiting current density of 5.8 mA/cm^2^ under alkaline conditions, ranking it among the finest noble metal-based ORR electrocatalysts reported. Furthermore, the resultant Pd_3_Cu aerogel exhibits superior performance in both the HER and the ethanol oxidation reaction (EOR). Moreover, DFT calculations indicate that the distinct partially oxidized Pd_3_Cu aerogel resulted in a downward shift in the d-band center of active sites, which energetically enhances the binding strength of adsorbed O intermediate during the ORR process, thereby accelerating ORR activity.

## 8. Benchmarking ORR Performance Across Gel-Derived Catalyst Types

**Figure 11:**
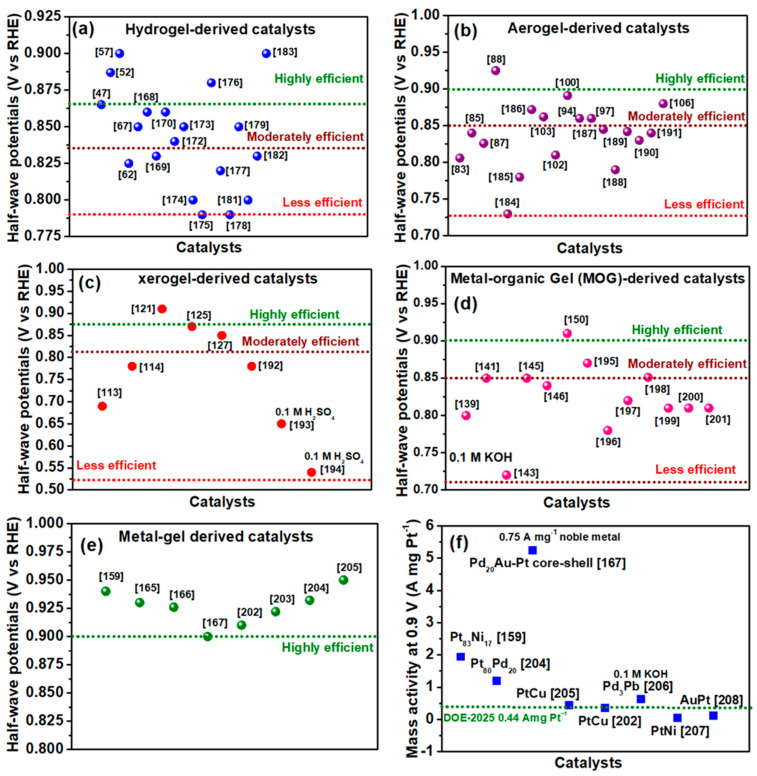
Comparison and classification of ORR activities of the catalysts derived from (**a**) hydrogels, (**b**) aerogels, (**c**) xerogels, (**d**) metal–organic gels, (**e**) metal gels, (**f**) and mass activities of various metal-gel catalysts.

We sought to find which kinds of gel-derived catalysts—including hydrogels, aerogels, xerogels, metal–organic gels (MOGs), and metal gels—show better overall performance after looking over a wide range of them. Table 2 shows the ORR kinetic data of several gel-derived catalysts that are collected from the literature for each category of gels, hydrogels [168,169,170,171,172,173,174,175,176,177,178,179,180,181,182,183], aerogels [184,185,186,187,188,189,190,191], xerogels [192,193,194], metal–organic gels [195,196,197,198,199,200,201], and metal gels [202,203,204,205,206,207,208]. Table 3 and Table 4 outline gel catalyst synthesis specifications, such as precursor ratio, gelation time, pyrolysis temperature, and Zn–air battery cycling performance of gel-derived electrocatalysts. Standard performance criteria must be chosen to make a meaningful comparison. Though mass and specific activities of the ORR catalysts are advised by the U.S. Department of Energy (DOE) as the main benchmarks for assessing catalyst efficiency, these measures are not regularly stated across all research papers. Actually, very few studies offer information on both mass and specific activities; thus, it is challenging to establish a universal basis for comparison from these criteria. We therefore chose the half-wave potential (E_1_/_2_) as the main assessment criterion to address this challenge. As a well-known parameter in ORR studies, the half-wave potential provides a consistent estimate of catalytic activity. Its general availability in the literature qualifies it as a suitable measure for comparison among several gel-derived catalyst forms. Figure 11 shows the relationship between the catalysts and their half-wave potentials. An arbitrary criterion for catalyst activity has been established: catalysts are categorized as “*highly efficient*” if their half-wave potentials are greater than 0.90 V vs. RHE, “*moderately efficient*” if they fall between 0.85 and 0.90 V vs. RHE, and “*less efficient*” if they fall below 0.85 V vs. RHE.

The catalysts made from metal gels or supportless catalysts only exhibit excellent electrocatalytic ORR activity according to the established criterion, and the majority of them cross the half-wave potential of 0.90 V vs. RHE. As a result, metal-gel catalysts are regarded as the best of all gel-derived catalysts. It should be mentioned, though, that all of these catalysts are made of precious metals and their alloys. Their high costs largely restrict commercial applications, despite the fact that they exhibit excellent ORR activities and mass activities that surpass the standard of 0.44 A mg Pt^−1^ set by the DoE 2025. Because of their high mass activity, they should perform better than commercial standards when used in a commercial cell, making them suitable for commercial applications. Metal-gel catalysts are regarded as the best catalysts for ORR in alkaline fuel cells like AEMFC and alkaline Zn–air batteries, as well as acidic fuel cells like PEM, if cost is not an issue.

Apart from metal gels, all other types of gel-based catalysts have been used to create transition metal-based catalysts, single-atom catalysts that are atomically dispersed, or metal-free/heteroatom-doped catalysts for ORR. These catalysts have primarily been studied in alkaline electrolytes (0.1 M KOH) and Zn–air battery applications. While xerogel- and MOG-derived catalysts demonstrated lower efficiencies for ORR, a number of hydrogen-derived catalysts, including aerogel catalysts, fall into the moderately active catalyst category with very few exceptions. From the above observations, the electrocatalytic ORR activity of a gel-derived catalyst can be ranked as outlined below.

In alkaline electrolytes, hydrogel and aerogel-derived catalysts are equally superior, thus ranked first, while xerogel- and metal–organic framework (MOF)-derived catalysts are ineffectual for ORR, consequently ranked second.

In acidic electrolytes, metal-gel catalysts exhibit exceptional activity for the ORR, with mass activities ranging from 4 to 18.7 times greater than those of the Pt/C catalyst, higher than the DoE target of 2025, establishing them as the leading catalysts in this category.

## 9. Key Insights

- After reviewing a number of research studies, it has been found that gel-derived catalysts show outstanding characteristics and electrocatalytic oxygen reduction reaction (ORR) activities, usually either equivalent to or better than those of commercial Pt/C catalysts.
- Exceptional porosity is a common and important characteristic of all five forms of gel-derived catalysts; it can be finely tuned from microporous to mesoporous and macroporous structures. These catalysts also have remarkably high specific surface areas—approaching or even surpassing 2000 m^2^ g^−1^, which surpasses traditional expectations.
- Achieving well-dispersed catalytic sites with minimum agglomeration depends especially on such high surface areas. This is particularly pertinent in the development of atomically scattered catalysts, where their high surface energy makes controlling the aggregation of single atomic sites intrinsically difficult.
- The abundance of surface area in gel-derived catalysts offers an ideal matrix to host a high density of single-atom catalytic sites without appreciable aggregation. On the other hand, attempts to include a high density of such sites into low-surface-area carbon supports usually lead to agglomeration.
- In particular, some studies have shown how effectively more than 15% of single-atom metallic active sites could be incorporated into gel-derived matrices without aggregation, so stressing their possible use as hosts for atomically distributed catalysts with high loading, without aggregations.
- In terms of the ORR activity, stability, porosity, and surface area among the several gel forms, hydrogels and aerogels have shown the most promise; metal–organic gels (MOGs) with a supramolecular assembly strategy and xerogels follow in second place of importance for synthesizing high densities of non-precious metal-based (M-N_4_-C, M = transition metal) ORR catalysts.
- For metal-aerogel-based catalysts, the proposed method has mostly produced the synthesis of noble metal alloy systems, especially supportless catalysts. These noble metal-based aerogel catalysts show remarkable electrocatalytic performance unlike transition metal-based catalysts with carbon supports, which are less commonly prepared via metal-aerogel routes. Aerogel-derived noble metal catalysts show remarkable mass and specific activities, according to several studies; in some cases, these values surpass the 2025 targets set by the U.S. Department of Energy for ORR performance. This emphasizes their great relevance for useful applications in systems of energy conversion. The scalability of these materials for mass production and their integration into useful devices like fuel cells and metal-air batteries still present major obstacles.
- For flexible Zn–air batteries, especially, it would be beneficial if these supportless metal-aerogel catalysts could be directly deposited onto flexible substrates. This would improve device commercial viability and streamline the manufacture of them. Generally speaking, aerogel-derived catalysts are a unique and quite promising class. They differ from other gel-derived catalysts in their unusual capacity to create supportless, noble metal alloy structures with extraordinary electrochemical performance. By means of ongoing research on scalable synthesis and flexible integration techniques, their acceptance in next-generation energy technologies could be accelerated.

## 10. Future Perspectives and Recommendations

- Although metal-aerogel catalysts have shown remarkably high oxygen reduction reaction (ORR) activity, their synthesis has been mainly limited to noble metals thus far, including platinum, palladium, and their alloys. Although these materials show great mass and specific activities—often exceeding DOE performance criteria—their great cost makes extensive commercial deployment very difficult. From an economic standpoint, then, noble metal-based aerogel catalysts are not seen as suitable for broad uses.
- A major development in ORR catalysis could come from metal-aerogel catalysts based on non-precious, earth-abundant transition metals. Given their cost-effectiveness and abundance, if such catalysts can attain mass and specific activities equivalent to their noble metal counterparts, they would present a quite appealing substitute. Moreover, the effective fabrication of non-precious metal-aerogel catalysts with high catalytic activities would open new paths for scalable production, making them feasible candidates for integration into fuel cell stacks and zinc–air batteries. Reaching this target will represent major progress in the design of next-generation, reasonably priced ORR electrocatalysts.
- Gel-based catalysts are appealing from scientific and practical perspectives due to their simple and effective synthesis paths. Precursor solutions often gel naturally and can be dried and turned into a functional catalyst without post-processing. This simplified process reduces material loss and chemical use by eliminating solvent-intensive washing, filtration, and purification. Gel-derived catalysts enable eco-friendly and affordable synthesis. Gel systems are more adaptable because they use cheap, readily available biomolecular precursors like gelatin, glycine, starch, alginate, dextrose, etc. These readily available components simplify and scale synthesis and enable gel development under moderate conditions. Gel-derived catalysts can be scaled up from laboratory to industrial levels using benign, low-cost precursors, allowing gram- to kilogram-scale production without compromising catalytic performance. Gel-based synthesis’s minimal processing, low solvent use, and sustainable precursors make it a promising electrocatalysis method.
- Several catalysts derived from hydrogels and aerogels exhibit extraordinary surface areas (approximately 2000 m^2^ g^−1^). However, when we attempted to establish a correlation between surface area and ORR activities, we did not observe any linear trends. Catalysts with the highest surface area exhibit comparable activities to those with a surface area of approximately 400 m^2^ g^−1^; therefore, we contend that a high surface area may not necessarily lead to enhanced ORR activity but facilitate the accommodation of substantial loads of metallic nanoparticles or single-atom catalysts without noticeable aggregation, which is typically challenging to achieve with conventional catalyst synthesis methods such as MOFs. Therefore, if high-surface-area catalysts can be utilized to incorporate elevated metallic loadings, it could lead to enhanced ORR and mass activities suitable for commercial applications.
- One of the primary limitations of ORR catalysts is the challenge in categorizing them as viable alternatives to Pt/C in practical fuel cells and Zn–air batteries, as only a limited number of catalysts report mass and specific activities, which are critical criteria for ORR catalysts. Due to the limitations of half-wave potential patterns, it is unlikely that their activities will be effectively translated into realistic energy storage and conversion devices. Consequently, it is recommended that the authors and other researchers also report on the mass and specific activities.
- Another limitation is that nearly 90% of the catalysts are exclusively evaluated in alkaline electrolytes, with only a few studies examining the catalytic activities in acidic electrolytes. Although the same catalysts can be utilized in both electrolytes, there is generally a substantial difference in their activities in acidic and alkaline electrolytes. On the other hand, ORR in basic electrolytes can be applied to AEM fuel cells and Zn–air batteries. PEM fuel cells, which are based on acidic conditions, are the most ideal for transportation applications, and it is essential to evaluate the ORR activity in acidic conditions and then principally in a single cell.
- The high surface area and hierarchical porosity within micro-/meso-/macropores of gel-derived catalysts comprise some of the best physicochemical properties that distinguish them from other conventional catalyst systems. Therefore, it is interesting to note the effect of the hierarchical porous structure on its pivotal role in facilitating an efficient mass flow of gases or transporting intermediate reactants, though modelling and experimental validation can solve one of the important issues in terms of catalyst thickness and ORR activity in a realistic Zn–air battery or fuel cells. It is very well known that non-precious metal catalysts require higher catalyst loading in order to deliver the desired power density, which, in turn, restricts the flow of O_2_ (especially when air is used as an oxidant). In this regard, gel-derived catalysts have been shown to possess balanced micro-/meso-/macropores that could reduce the mass transport resistance associated with the O_2_ diffusion from the bulk to the catalyst layer and quick H_2_O removal.
- Carbonaceous frameworks made from gel-based materials without metallic active sites or heteroatom doping can be used as gas diffusion layers (GDLs) in electrochemical energy devices, as well as active electrocatalysts or catalyst supports. These gel-derived carbons naturally have hierarchical porous architectures with interconnected micropores and mesopores for fuel cell and metal–air battery gas transport and electrolyte access. Mesopores mimic conventional GDL materials’ ideal structures by increasing gas permeability and lowering mass transfer resistance, while micropores increase surface area and capillary condensation. By creating materials in desired shapes and thicknesses, the direct gel-to-carbon conversion technique allows GDL design and integration flexibility. Due to their low cost, scalability, and tunable porosity, these metal-free, heteroatom-free gel-derived carbons could replace commercial GDLs made from carbon cloths or carbon papers in next-generation flexible or portable electrochemical devices.
- Among different types of gels, hydrogels offer unique possibilities of synthesizing electrocatalysts from biological sources like gelatine, agar, starch, cellulose, alginate, hyaluronic acid, etc., which not only make them sustainable precursors but also impact the catalyst synthesis cost. Therefore, we recommend that more research should be conducted on hydrogel-derived catalysts for SAC synthesis and further translate the synthesis process from the lab scale to the gram level.
- Among all types of gels, the precursor toxicity is the lowest for hydrogel-derived catalysts due to the use of natural gelling agents, whereas in all other catalysts, a specific organic ligand is used that is either expensive or toxic to the environment.
- In terms of scalability, hydrogel-derived catalysts have tremendous potential, followed by xerogels and metal–organic gels. In contrast, aerogels and metal gels possess specific challenges in scalability due to their complexity in the synthesis and post-synthesis processes, such as freeze-drying or supercritical drying, and sensitivity to pH, temperature, and the use of structural directing agents.
- So far, in the hydrogel-derived catalysts, the use of heteroatom-containing ligands has not been established. It is important to note that natural gelling agents intrinsically possess some heteroatoms; however, introducing high concentrations of the different heteroatoms could synergistically improve the ORR activity of SACs. In addition, high concentrations of heteroatoms, such as N, improve the electronic conductivity and high density of M-N_4_-C active sites. Furthermore, other dopants such as S, P, B, and F can further optimize the polarization of the carbon matrix, which benefits from enhanced ORR activity. Therefore, we recommend the modification of the hydrogel’s synthesis by introducing heteroatom-containing ligands as novel gel synthesis routes.
- The hydrogel- and aerogel-derived catalysts could possess extremely high BET surface areas > 1000 m^2^ g^−1^; therefore, these catalysts possess extremely high possibilities of introducing high loading SACs. Therefore, we recommend exploring the hydrogel synthesis catalysts for high loading SAC studies that can be game-changing by achieving the high mass and specific activities set by DoE.
- At present, the xerogel-derived catalysts are found to be the least active in ORR. Therefore, we recommend the hybridization of xerogels with other 2D/3D advanced materials like MOF/COF/MXenes to create a hybrid catalyst for improved ORR kinetics.
- In terms of MOG catalysts, there is a need to develop alternatives/explore high-coordinating ligands to improve the coordination environment and gelation kinetics.
- One of the highly possible and anticipated research areas includes the development of metal aerogels that are made of non-precious/transition metals such as Fe, Co, Ni, and Mn. The noble metal catalysts have already evidently shown extraordinary mass and specific activities. However, metal aerogels from non-precious/transition metals such as Fe, Co, Ni, and Mn have rarely been synthesized and explored for ORR catalysts. Therefore, we highly recommend future research to explore non-noble metal aerogel catalysts for ORR catalysis.

In conclusion, gel materials demonstrate potential for advanced energy conversion applications such as Zn–air batteries and fuel cells. They are appealing for their tunable properties, hierarchical porous frameworks, high surface area, scalability, cost-effectiveness, and excellent electrochemical ORR activity. The inherent stretchability, flexibility, and bending abilities of gel-derived catalysts maintain high performance, good rate capabilities, electrochemical stability in both acidic and alkaline electrolytes, dominant four-electron reduction reactions, and high densities of single-atom catalysts such as Fe-N_4_-C/Co-N_4_-C. Overall, gel materials’ diverse and beneficial properties indicate their potential to advance energy technologies.

## Figures and Tables

**Table 1:** Classification of various gels and their characteristics, synthetic routes, advantages, and limitations in ORRs.

Type of Gel	Characteristics	Synthesis Route and Scalability	Advantages	Limitations
Hydrogel	High water content Gel contains 3D network Enhances the metal/SACs distribution	Polymerization in the presence of metal precursors, followed by cross-linking Facile doping of heteroatoms Scalability: Very high	High BET surface area High micro-/meso-/macroporosity Low cost of the precursors and eco-friendly precursors Excellent distribution of M-N_4_-C active sites Moderate to high ORR activity	Pre-drying is required before pyrolysis
Aerogel	Excellent retention of the porosity Ultra-lightweight and very high surface area Excellent combination of 3D meso-/macroporosity	Gel formation from solution (Sol–gel) → freeze/supercritical drying Scalability: Moderate to High	Excellent porosity of the catalysts Extremely high surface area Possibility of high loading of SACs without aggregation Excellent distribution of M-N_4_-C active sites High ORR activity	High-cost instruments used for freeze/supercritical drying Time consuming and energy intensive
Xerogel	Poor retention of the porosity	Gel formation from solution (Sol–gel) → hot air oven drying Scalability: High	Low cost Simple drying of the gel Moderate to low ORR activity	Disintegrated porosity not suitable for electrocatalysis Poor dispersion of metal NPs
Metal–organic gel (MOG)	Hybrid catalysts synthesized from organic ligand/polymer with metal salt precursors	Coordination reaction with organic ligands and metal precursors to form gel Scalability: Moderate to high	Easy to synthesize Versatility of the catalyst synthesis Often leads to agglomeration of NPs with organic ligands Even dispersion of SACs and M-N_4_-C active sites when coordination polymers are used Moderate to high ORR activity	pH dependence on the kinetics of gelation
Metal aerogel	Only metallic nanoparticles network Support less catalysts 3D network of porosity	Simple reduction in the metallic precursors with reducing agents Scalability: Moderate to low	Extremely high mass and specific activity Excellent stability High to very high ORR activity	Complex synthesis steps High cost of the catalyst

**Table 2:** ORR kinetic data, Zn–air battery performance of several gel-derived catalysts that are collected from the literature for each category of gels, hydrogels, aerogels, xerogels, metal–organic gels, and metal gels.

Catalyst	^a^ Surface Area (m^2^ g^−1)^ ^b^ Porosity (nm) ^c^ Pore Volume (cm^3^/g)	ORR Active Site	Half-Wave Potentials (V vs. RHE) 0.1 M KOH ^a^ 0.1 M HClO_4_^b^ Pt/C Standard ^c^	^a^ Tafel Slope (mV dec^−1^) ^b^ No. of Electrons from K-L and RRDE ^c^ % of H_2_O_2_/H_2_O^−^	^a^ Number of Potential Cycles/loss in E_1/2_ (mV) ^b^ Chrono-Amperometry Current Retention	^a^ Fuel Cell ^b^ Zn–Air Battery Performance ^c^ Specific Capacity	Ref.
Hydrogel-derived catalysts							
HP/FeCo-NC-2	^a^ 771, ^b^ 4 nm	Atomically dispersed Fe/C-N_x_	^a^ 0.8651 ^c^ 0.86	^a^ 95 ^b^ 23 ^c^ 3.87−3.94, ^d^ 7%	^b^ 90%/15 h	NR	[47]
CuFe AC@NC	^a^ 289, ^b^ 4 nm	Fe-N_4_-C and Co-N_4_-C	^a^ 0.887 ^c^ 0.853	^a^ 78 ^b^ 3.83–3.91	^b^ 95%/12,000 s	^c^ 806.2 mAh g^−1^	[52]
Fe_2_N/NC-1	^a^ 216	Fe_2_N NPs @NC	^a^ 0.90 & 1.01 with NH_3_ treatment ^c^ 0.85	^a^ 102 ^b^ 3.81/3.91–3.99 ^c^ 5%	NR	NR	[53]
Co−N−C-0.02	^a^ 493 ^c^ 1.49–1.60	CoN_4_-C	^a^ 0.825 ^b^ 0.691 ^c^ 0.825	^a^ 36 ^b^ 3.65−4.00 ^c^ ≈17.4–0.00%	^a^ 5000/11 ^b^ 25 h/72%	NR	[63]
CPP-900	^a^ 1002 ^b^ 0.837	N, P doping Fe SACs	^a^ 0.848 ^c^ 0.982	^a^ 81 ^b^ 3.94, ^c^ <1.72%	^b^ 20,000/91%	^b^ 204 mW cm^−2^ ^c^ 811 mAh g^Zn−1^	[54]
NPMC-1000	^a^ 1663 ^b^ <10 nm ^c^ 1.10	N and P dopants	^a^ 0.85	^b^ ∼4.0/3.85 ^c^ 8%	NR	^b^ 55 mW cm^−2c^ 735 mAh g^−1^ @5 mA cm^−2^	[67]
C-Fe-UFR	^a^ 433 ^b^ 1.144	Metallic Fe and Fe-N_x_	^a^ 0.86	^b^ 3.93–3.98 ^c^ 6%	^a^ 10,000/22	^b^ 142 mW cm^−2^ ^c^ 467 mAh g^−1^ @5 mA cm^−2^	[168]
PANI-EN- hydrogel	^a^ 1400 ^b^ <2	Fe-N_4_	^b^ 0.83	^a^ 118 ^b^ ~4, ^c^ <1%	^a^ 10,000/14	NR	[169]
Ppy/FeTCPP/Co	^a^ 472 ^b^ micro- and mesopores	Fe–N–C Co–N_x_–C and Co ^o^	^a^ 0.86 ^b^ 0.72 ^c^ 0.82	^a^ 61 ^b^ 4/3.93 ^c^ 2%	^b^ 95%/10 h	Rechargeable Zn–air battery round-trip efficiency 62%	[170]
PtFeCo/GCM	^a^ 728 ^b^ 5.6 ^c^ 0.65	PtFeCo alloy	^b^ 0.916	NR	^a^ 20,000/25	NR	[171]
PANI-Fe/PA -N1050	NR	N doping and Fe-N_x_	^a^ 0.84 ^c^ 0.88	^b^ 3.3	^b^ 1000/14	NR	[172]
CoFe-PPy	NR	N doping and CoN_x,_ and Fe	^a^ ~0.85 ^c^ ~0.85	^a^ 60 ^b^ ~4	5000 0.85	NR	[173]
CoFe@N-CNWF	^a^ 233 Mesopores	N doping and Fe and Co	^a^ 0.80 ^c^ 0.84	^b^ 3.68–3.87 ^c^ <15%	^b^ 20,000 s/11.7%	^b^ 90 mW cm^−2^ ^c^ 806 mA hg_Zn_^−1^	[174]
NiPcTs/Co/Py	NR	N doping, Ni and Co	^a^ ~0.79 ^c^ ~0.80	^b^ 3.83	^b^ 35,000 s/75%	NR	[175]
CoO_x_/Co−N−C (800)	^a^ 786 ^b^ 2–6 nm ^c^ 0.144	N doping, Co_x_ and Co-N_x_	^a^ 0.88 ^c^ 0.88	^a^ 61.7 ^b^ 3.97/3.80 ^c^ <10%	^b^ 20,000 s/83.8	NR	[176]
P(AA-AM)(5-1)-Co-N	^a^ 1397	N doping and Co–N_x_/C	^b^ 0.820 ^c^ 0.854	^a^ 60.8 ^b^ ~3.9 ^c^ 17%	^a^ 5000/4	^a^ 0.66 W cm^−2^ (H_2_-O_2_) 0.28 (H_2_-Air)	[177]
PF-800	^a^ 370 ^b^ 0.5, 5.4, ^c^ 0.76	N doping, Fe and Fe-N_x_	^a^ 0.79	^b^ 3.75–3.99 ^c^ 9.1	^b^ 20,000 s/89%	^b^ 131 mW cm^−2^ ^c^ 748 mAh g_Zn_^−1^	[178]
FeCo/FeCoP@ NP-CF	542 ^a^ 3.98	FeCo, Fe_2_P, Co_2_P	^a^ 0.85 ^c^ 0.84	^a^ 107 ^b^ 3.98	^b^ 15 h/91%	NR	[179]
Fe- Ni-NC	NR	N-doping, Ni and FeN_x_	^a^ 0.66	^a^ 93.2 ^b^ 3.93 ^c^ 3.31%	^b^ 1000 s/81%	NR	[180]
CNS-900	NR	N and S doping	^a^ 0.80 ^c^ 0.823	^a^ 37 ^b^ 3.9	^a^ 5000/16	NR	[181]
Co_2_P/H-NPC	^a^ 208 ^b^ 19.6 ^c^ 0.15	N,P doping Co-O, Co-P	^a^ 0.83	^a^ 47 ^b^ 3.7	^a^ 10,000/49	^b^ 120 mW cm^−2^ ^c^ 847 mAh g_Zn_^−1^	[182]
Fe/Fe_3_C@Fe-N_x_-C-950	^a^ 535 ^b^ 5–50 nm	N doping Fe_3_C, FeN_x_-C	^a^ 0.90	^a^ 56 ^b^ 3.74/^c^ <3%	^b^ 40,000 s/no loss	^b^ 120 mW cm^−2^	[183]
Aerogel-derived catalysts							
Fe─N─C/TiN	^a^ 540 ^b^ 5, 10, 50	Fe SACs TiN	^b^ 0.806	^b^ 4 ^c^ 1–4%	^a^ 30,000/15	^a^ H_2_─O_2_ 0.90 W cm^−2^	[68]
(NiFe-LDH)_n_/GA_x_	^a^ 344 ^b^ <2	N doping Ni^2+^ to Fe^3+^	^a^ 0.840 ^c^ 0.831	^a^ 78 ^b^ 3.97/^c^ 6.4%	^a^ 5000/8	^b^ 230 mW cm^−2^ ^c^ 49 mAh g_zn_^−1^	[85]
Fe-NBrGO	^a^ 553 ^b^ 2–4	B and N Fe_3_O_4_, Fe_3_C	^a^ 0.826	^b^ 3.8	NR	^a^ 107 mW cm^−2^	[87]
Pd_3_Cu@NC	^a^ 96	Pd_3_Cu alloy NPs	^a^ 0.925	^a^ 90 ^b^ 4.12 ^c^ 2–3%	NR	NR	[88]
HT800-FeP	NR	N and Fe-N_4_ SACs	^a^ 0.86	NR	NR	^a^ 580 mW cm^−2^ H_2_-O_2_ AEMFC	[94]
Fe-N/P/C-850	^a^ 615 ^b^ 0.52	N, P doping Fe-N_x_	^a^ 0.86 ^c^ 0.84	^a^ 64.7	^b^ 30,000/95.5%	NR	[97]
Fe–Ni ANC@NSCA	^a^ 241 ^c^ 0.24	N,S doping Fe-N_x_, Ni-N_x_,	^a^ 0.891 ^c^ 0.876	^a^ 70 ^c^ 4	^a^ 10,000/no loss	^b^ 140 mW cm^−2^ ^c^ 750 mA.h.g_Zn_^−1^	[100]
FeCo/N-DNC	^a^ 260	N doping Fe-N_x_	^a^ 0.81 ^c^ 0.84	^b^ 3.92	^b^ 10,000 s/19.7%	^b^ 115 mW cm^−2^ ^c^ 804 mA.h.g_Zn_^−1^	[102]
Pd_3_Pb/rGO-CNTs aerogel	^a^ 134 ^b^ 22–50	Pd^0^/Pd^2+^ Pb^0^/Pb^2+^	^b^ 0.862 ^c^ 0.841	^b^ 3.84/^c^ 8%	^b^ 10,000/17.6%	NR	[103]
S-C_2_ NA	^a^ 1943 ^b^ 3 nm ^c^ 1.56	N, P and S doping	^a^ 0.88 ^c^ 0.85	^a^ 54 ^b^ 3.98 to 4.02/^c^ 6%	^a^ 5000/no loss ^b^ 10 h/no loss	^b^ 209 mW cm^−2^ ^c^ 863 mA.h.g_Zn_^−1^	[106]
Co−N−GA	^a^ 485 ^c^ 0.71	N doping Co and Co-N	^a^ 0.73 ^b^ 0.85	^b^ 3.75−3.85/13 ^b^ 3.94−3.97/2.26	^a^ 5000/15	NR	[184]
Ni-MnO/rGO	^a^ 109 ^b^ 13.5	Mn^2+^/Mn Ni^2+^/Ni	^a^ 0.78 ^c^ 0.84	^b^ 85 ^c^ 4	^b^ 10,000/93%	^b^ 123 mW cm^−2^ ^c^ 758m A.h.g_Zn_^−1^	[185]
CoO_x_/NG-A	^a^ 814 ^b^ 5	N doping and CoO_x_	^a^ 0.872	^b^ 3.8	^a^ 3000/26	NR	[186]
N, B, F@Co-CNF	^a^ 718	N, B, F Co-N_x_	^a^ 0.845 ^c^ 0.834	^a^ 69	^b^ 20,000/85%	NR	[187]
Fe-N-C aerogel	^a^ 292	N doping Fe-N_x_	^a^ 0.79	^a^ 92 ^b^ 4/^c^ 2%	NR	NR	[188]
Ce/Fe/NCG-2	^a^ 699 ^b^ 2–7 nm	N doping Fe-N_x_	^a^ 0.842 ^c^ 0.857	^a^ 58.4 ^b^ ~4	^a^ 3000/24	100.7	[189]
GH-N-C-900	^a^ 786 ^c^ 0.76	N doping	^a^ 0.830	^b^ 3.53/3.58–3.82 ^c^ 20%	NR	NR	[190]
N-GA-4-900	^a^ 205 ^c^ 0.278	N doping	^a^ 0.84 ^c^ 0.84	^a^ 92.5 ^b^ 3.98	^b^ 18,000 s/92%	NR	[191]
Xerogel-derived catalysts							
MnO/N-CC-2-900-2	^a^ 259 ^b^ 3.28	N doping Mn-O	^b^ 0.69	^b^ 3.94	^a^ 20,000/10	NR	[113]
MnO/N-CC-5	NR	N doping Mn-O	^a^ 0.78 ^b^ 0.81	^a^ 150 ^b^ 3.95	^a^ 5000/10 ^b^ 25 h/97.5	NR	[114]
ISG Fe-N-C	^a^ 704 ^b^ 3.2	Fe-N_x_ Fe SACs	^a^ 0.91 ^b^ 0.74 ^c^ 0.85	^a^ 64 ^b^ 4 ^c^ <5%	^a^ 5000/8 ^b^ 50,000/93%	^b^ 259 mW cm^−2^ ^c^ 763 mA.h.g_Zn_^−1^	[121]
Fe-Ac-2	^a^ 950 ^c^ 0.77	N doping Fe-N_x_	^a^ 0.87 ^c^ 0.85	^a^ 81	^a^ 12 h/94%	153	[122]
Co_9_S_8_@NS-C	^a^ 409	N, S doping Co-O/Co-S	^a^ 0.85 ^c^ 0.87	^a^ 3.84–3.98	^b^ 36,000 s/94%	NR	[123]
CoNC@NCXS-800	NR	N doping and CoN_x_	^a^ 0.78 ^c^ 0.80	^a^ 137 ^b^ 3.9 ^c^ <15%	^a^ 1000/21	^b^ 67 mW cm^−2^ ^c^ 710 mA.h.g_Zn_^−1^	[192]
Fe-N-CXG-H_2_O	^a^ 1267 ^b^ 1.15 ^c^ 0.54	N doping, Fe-N_x_	^b^ 0.65 ^c^ 0.820.54	^a^ 51 ^b^ 4.0	56%, Current loss after 20 h at 0.5 V in fuel cell	^a^ 200 mWcm^−2^	[193]
Fe-N-CXG-5.8-2-T2	^a^ 445^b^ 8.8 ^c^ 0.45	N doping, Fe-N_x_	^b^ 0.54	^a^ 75 ^b^ 3.53	NR	NR	[194]
Metal–organic gel-derived catalysts							
P-CoFe-H3	^a^ 89 ^b^ 3.7	N, P doping Co-N_x,_ Fe-N_x_	^a^ 0.80 ^c^ 0.86	NR	NR	^b^ 98 mW cm^−2^	[139]
CoP@NPCA-900	^a^ 683 ^c^ 1.44	N, P doping Co-P	^a^ 0.85	^b^ 3.99	NR	^b^ 125 mW cm^−2^ ^c^ 668 mA.h.g_Zn_^−1^	[141]
Fe-MOG-MFN-C	^a^ 950 ^c^ 0.10	N and Fe-N^x^	^a^ 0.91(onset) ^c^ 0.91(onset)	^a^ 68.5 ^b^ 3.6/^c^ 20%	^a^ 5000/31	NR	[142]
MOG(Fe)/urea/CNTs-700	^a^ 150 ^c^ 0.27	N, Fe and FeNx	^a^ 0.72	^a^ 51 ^b^ 3.51–3.92 ^c^ <25%	^b^ 20,000/91.7%	NR	[143]
Co@N-PCP/NB-CNF-2-800	^a^ 228 ^b^ 5.8	N, B doping Co and Co-N_x_	^a^ 0.85 ^c^ 0.83	^a^ 68.28 ^b^ 3.7 ^c^ <10%	^a^ 10,000/24	^b^ 143.8 mW cm^−2^ ^c^ 700 mA.h.g_Zn_^−1^	[144]
NiFe/B,N-CNFs	^a^ 125	N doping, Fe, Ni-N_x_	^a^ 0.84 ^c^ 0.82	^a^ 3.77	NR	^b^ 159 mW cm^−2^ 137h stable	[145]
SA-Fe-N-2-800	^a^ 1007 ^b^ 2–100	N doping and Fe SACs	^a^ 0.910 ^b^ 0.812	^a^ 72 ^b^ 3.9, ^c^ ~5%	^a^ 5000/10	NR	[150]
PON/C-“Rb”	^a^ 1380	N and P doping	^a^ 0.87 ^c^ 0.83	^b^ 3.93–3.95 ^c^ <5%	^b^ 20 h/85%	^c^ 705 mA.h.g_Zn_^−1^	[195]
CHI-TMA-Fe-CW-M1	^a^ 565	N and Fe-N, Fe_2_O_3_	^a^ 0.78 ^c^ 0.83	^a^ 90.9 ^b^ 3.8 ^c^ 7.8–13.8	^a^ 5000/24	NR	[196]
Co/N@PCS-900-1	^a^ 742 ^c^ 0.445	N, doping Co	^a^ 0.82 ^c^ 0.79	^b^ 3.98~4.00	^b^ 50,000 s/94.7%	NR	[197]
CoNC-MOG-9	^a^ 351	N doping and CoN_x_	^a^ 0.851 ^c^ 0.83	^a^ 78 ^b^ 3.92 ^c^ <15%	^a^ 5000/no loss	63	[198]
CoO@Co@N/C	NR	N doping Co, CoO	^a^ 0.81 ^b^ 0.83	^b^ ~3.9 ^c^ ~5%	^b^ 20,000/98%	NR	[199]
Co2P@CoNPG-900	^a^ 93.8 ^b^ 2.8 ^c^ 0.258	N doping Co-N_x_, Co-O	^a^ 0.81 ^c^ 0.82	^a^ 69 ^b^ 3.96	^b^ 12,000/91.6%	NR	[200]
Co_2_P/C	NR	N, P doping Co-P	^a^ ~0.81	^b^ ~4 ^c^ <20%	^b^ 20,000/no loss	NR	[201]
Metal-gel-derived catalysts							
Pt_83_Ni_17_ BNCs AGs/C	^a^ 58.4 ^b^ 5–7	Pt-Ni alloy	^b^ 0.94 ^c^ 0.89	^b^ ~4	^a^ 20,000/6.1	NR	[153]
Pd_3_CuFe_0.5_	^a^ 75 ^b^ 15.29	Pd-Cu-Fe alloy	^a^ 0.93 ^b^ 0.86	^a^ 96 ^b^ ~4	^b^ 16,000/95%	^b^ 93.2 mW cm^−2^	[165]
PtCu aerogel	^a^ 43.6	PtCu alloy	^b^ 0.926 ^c^ 0.888	NR	^a^ 5000/20	NR	[166]
Pd_3_Cu aerogel	^a^ 44 ^b^ 8.77	PdCu alloy	^a^ 0.90 ^b^ 0.85	^a^ 50	^b^ 1700/13	NR	[167]
Au-Pt aerogel	^a^ 95.8 ^c^ 0.339–0.640	Au-Pt alloy	^a^ 0.91 ^b^ 0.86	^a^ 73 ^b^ 3.9–4.0 ^c^ 1–4%	^a^ 1000/12 (0.1 M KOH ^b^ 1000/9 (0.1 M HClO_4_)	NR	[202]
Pd_20_Au aerogel	^a^ 83–105	PdAu alloy	^b^ 0.922	^b^ 4	^a^ 10,000/no loss	NR	[203]
PtCu aerogel	NR	PtCu alloy	^b^ 0.932 ^c^ 0.865	^b^ 4 ^c^ <1%	^a^ 30,000/no loss	NR	[204]
IM-Pd_3_Pb NNs	^a^ 23.3	PdPb alloy	^a^ 0.95	^a^ 56.3 ^b^ ~4	^b^ 10,000/16	NR	[205]

**Table 3:** Gel catalyst synthesis specifications such as precursor ratio, gelation time, and pyrolysis temperature.

Catalyst	Precursor Ratio	Gelation Time	Pyrolysis Temperature (°C)—Time	Ref.
Hydrogels				
HP/FeCo-NC-2	Fe:Co:Melamine:Salicylic acid:2-Methylimidazole:Zn = 1.5:1:41:45:70:51	30 min	950—2 h	[47]
CuFe AC@NC	Glutamic acid:Fe:Chitoson:Cu = 3.2:1.8:5.3:1	~5 min	900 °C—2 h	[52]
Fe_2_N/NC-1	GO:Heme = 2.6:1	~12 h (hydrothermal)	900 °C, 1 h, N_2_ + NH_3_, 5 °C/min	[53]
Co−N−C-0.02	Polypyrrole:SDS:APS:Co(acac)_3_ = 3.6:5:12:1	12 h (polymerization)	800 °C/2 h Before, after acid leaching	[63]
NPMC-1000	Aniline: Phytic acid:Ammonium persulphate: 5 mL:20 mL:0.96 g	Overnight	1000 °C/2 h	[67]
C-Fe-UFR	Fe:Formaldehyde:Urea: 1.21 g:3.6 mL:1.8 g	20 s	900 °C/1 h	[168]
PANI-EN- hydrogel	Aniline:APS:FeCl_3_: 3.54:3.54:7.1 (mmol)	20 min	900 °C, 1 h Before, after acid leaching	[169]
Ppy/FeTCPP/Co	Pyrrole:FeTCPP:NaOH:APS:Co(NO_3_)_2_ (immersed in): 42 µL:14 mg FeTCPP:2.7 mg:137 mg:0.1 M	Instantly	800 °C for 4 h	[170]
PANI-Fe/PA -N1050	Aniline:FeCl_3_:pyretic acid: ammonium peroxysulfate (APS): 450 µL:20 mg:50 µL:286 mg	After several minutes	1050/2 h	[172]
CoFe-PPy	Pyrrole:Co(II)(bpdc)3 (or Fe(II)(bpdc)3:APS: 42 µL:1 mL:0.6 mmol	2 h	800 °C for 4 h	[173]
NiPcTs/Co/Py	Pyrrole:APS:NiPcTs:Co (NO_3_)_2_: 42 µL:0.137 g:0.0154 g:0.1 M Co (NO_3_)_2_	Instantly, hydrogel was immersed in Co^2+^ ions for 48 h	800 °C for 4 h	[175]
CoOx/Co−N−C (800)	CoPc:Chitosan:acq. GO solution: 0.06 g:3% (*w*/*v*):5 mg/mL	Overnight	800 °C for 2 h	[176]
P(AA-AM)(5-1)-Co-N	Acrylic acid:APS:BIS:CoCl_2_:cyanamide: 0.95 mL:0.19:0.2:2.8 mL	2 h	800 °C for 1 h	[177]
FeCo/FeCoP@ NP-CF	Fe:Co:PAM:pyritic acid (PA):melamine: 1.39:1.0:0.24:0.45:3.27	24 h	800 °C for 2 h	[179]
Fe- Ni-NC	Agar:acrylamide:MBAA (cross linker):Irgacure 2959 (Initiator):Fe:Ni: 0.18:1.00:0.0018:0.064:0.5 M:0.5 M	30 min @ 4 °C	800 °C for 1.5 h	[180]
Co_2_P/H-NPC	Polyinosinic acid:starch:NH_4_Cl:Co acetate: 1.00:4.00:3.00:0.25	5 min @ 110 °C	900 °C for 2 h	[182]
Fe/Fe_3_C@Fe-N_x_-C-950	Fe:EDTA: 0.550 g:1.9 g Fe-EDTA (complex):Glucose:NaNO_3_:Melamine: 1.00:7.20:8.40:10.08	Following RT mixing and stirring	950 °C for 2 h	[183]
Aerogels				
Fe─N─C/TiN	Resorcinol:Formaldehyde:TiO_2_ sol:Fe:propylene oxide: 1.00:0.57:1.96:0.098:0.204	5 h at 60 °C	950 °C for 1 h and NH_3_ gas	[68]
(NiFe-DH)_n_/GA_x_	Gelation:Ni:Fe: 2.5 g:1 M:1 M	2 h @ 4 °C	900 °C for 2 h	[85]
Fe-NBrGO	GO:Urea:Boric acid:Iron nitrate:NH_3_ solution: 1.00:20.83:4.17:0.60	12 h—autoclave @ 180 °C	900 °C for 2 h	[87]
Pd_3_Cu@NC	(i) Resorcinol:Urea:Formaldehyde: 1:0.24:2.13 → NC gel (ii) Pd:Cu:Na_2_CO_3_, Glyoxylic acid:NC: 3:1:371 mg:46 mg	Not Specified	(i) 900 °C for 2 h (ii) No pyrolysis	[88]
Co−N−GA	GO solution:Co:PANI: 2 mg mL^−1^:15 mg:80 mg	12 h at 180 °C—hydrothermal	900 °C for 1 h	[184]
Ni–MnO/rGO Aerogels	Mn:Ni:GO:PVA: 6.0 mg:25.8 mg:8 mg mL^−1^:16 mg mL^−1^	5 min	600 °C (10% H_2_/Ar)—15 h	[185]
Pd_3_Pb/rGO-CNTs	Pd:Pb:GO:CNTs:PVA: 8.0 mg:5.0 mg:4 mg mL^−1^:4 mg mL^−1^:16 mg mL^−1^	5 min	600 °C (10% H_2_/Ar)—12 h	[103]
FeCo/NDNC aerogels	K_4_Fe(CN)_6_:K_3_Co(CN)_6_:Chitosan:GO: 1 mL (0.5 mmol):1 mL (0.5 mmol):10 mg mL^−1^), 20 mg	5 min	600 °C for 3 h H_2_	[102]
Fe–Ni ANC@NSCA	Fe:Ni:aniline:Tannic acid:APS: 1:2.47:9.38:0.69:0.63:15	12 h	800 °C for 3 h	[100]
Xerogels				
MnO/N-CC-2-900-2	KMnO_4_:glucose:melamine:N-doped carbon: 1.0 g:4.78 g:1 g:100 mg	2–3 min	900 °C—1 h	[113]
MnO/N-CC-5	KMnO_4_:glucose:melamine:N-doped carbon: 1.0 g:4.78 g:1 g:400 mg	2–3 min	900 °C—1 h	[114]
ISG Fe-N-C	Glucosamine-HCL:ferrous gluconate:ammonia:TEOS: 1.5 g:0.75 g:50 µL:10 mL	3 h at 60 °C	900 °C—2 h	[121]
Fe-AC-2	FeCl_3_:agarose:activated carbon: 1:3:3	2 h at 70 °C	800 °C for 2 h	[122]
gl45-900	Co:Mg:thiourea:glycise: 5 mmol:15 mmol:10 mmol:45 mmol	Not Specified	900 °C for 2 h	[123]
CoNC@NCXS-800	(i) NH_3_:ethanol:H_2_O:resorcinol: 0.1 mL:8 mL:20 mL:0.2 g (ii) (i) + ZIF-67:formaldehyde: 0.5 g:0.28 mL	(i) 30 min (ii) 100 °C—24 h (hydrothermal)	800 °C for 2 h	[192]
Metal–Organic Gel				
P-CoFe-H_3_	Co:Fe:Phytic acid:H_3_TATAB: 20 mM:20 mM:528 mg:20 mM	30 min at 80 °C	Not Specified	[139]
N_3_/Fe/C-Pt	2-aminopyridine:Fe: 0.1g mL^−1^:50 mg mL^−1^	Not Specified	900 °C for 2 h	[140]
Zn_0.90_Co_0.10_-BMOG	GMP:ZnCl_2_:CoCl_2_: 2.04 g:0.90 mM:0.10 mM	5 min stirring stand still—12 h	900 °C for 2 h	[141]
Fe-MOG-MF IPN	Trimesic acid:FeCl_3_: 1:3 melamine and formaldehyde: 1:3 naphthalene (10% *w*/*w*)	overnight	900 °C for 3 h	[142]
MOG(Fe)/urea/CNTs-700	Trimesic acid:Fe:CNTs:Urea: 1:1:75 mg:125 mg	several seconds	700 °C for 5 h	[143]
CoNC-MOG-9	FA-Co gels:Folic acid:NaOH:CoCl_2_:NaCl: 530 mg:96 mg:142.2 mg:5 g	5 min stirring stand still—4 h	900 °C for 1 h	[198]
NiFe/B,N-CNFs	ptpy-B(OH)_2_:melamine:guanosine:K_3_Fe[(CN)_6_]–Ni(NO_3_)_2_: 24.7 mg:20 mg: 0 mg:1:1 molar ratio	5 min	900 °C for 2 h	[145]
SA-Fe-N	Sodium alginate:FeCl_3_:cynamide:certain amount: 1 g:1.0 mL (50% solution)	3 h at 60 °C	800 °C for 1 h	[150]
Metal gels				
Pt_83_Ni_17_ BNCs AG	5 mL of 18-mM NiCl_2_ and 67 μL of 0.445-M H_2_PtCl_6_, 60-mM NaBH_4_	10 min	No pyrolysis	[153]
Pd_3_CuFe_0.5_	Na_2_CO_3_ (0.2968 g), glycolic acid monohydrate (0.0368 g), PdCl_2_ (3 mm), CuCl_2_·2H_2_O (1 mm), and FeCl_3_·6H_2_O (0.5 mm)	10 min at 60 °C	No pyrolysis	[165]
PtCu	Na_2_PtCl_4_ (685 μL, 5 × 10^−5^ mol) CuCl_2_ (200 μL, 5 × 10^−5^ mol) NaBH_4_ (5 mL, 2 × 10^−3^ mol)	Several seconds	No pyrolysis	[166]
Au–Pt	Trisodium citrate dehydrate (400 × 10^−3^ M, 25 μL), HAuCl_4_·3H_2_O (32.5 × 10^−3^ M, 15.4 μL), K_2_PtCl_4_ (32.5 × 10^−3^ m, 15.4 μL), and NaBH_4_ (200 × 10^−3^ m, 20 μL) NH_4_F (1 M, 555 μL)	~6 h	No pyrolysis	[202]
PtCu	70 μL CuCl_2_·2H_2_O (0.6 mol), 2 mL H_2_PtCl_6_·6H_2_O (20 mmol), 10 mg NaOH, 5 mL NMP	180 °C for 8 h autoclave	No pyrolysis	[204]

**Table 4:** Zn–air battery cycling performance of gel-derived electrocatalysts.

Catalyst	Zn–Air Battery Cycling Performance	Ref.
CuFe AC@NC	Galvanostatic charge–discharge research demonstrated that ZABs maintain a steady voltage gap of 0.80 V for approximately 450 cycles, with each cycle lasting 10 min and a current density of 5 mA cm^−2^.	[52]
C-Fe-UFR	The discharge–charge testing of Zn–air batteries at 10 mA·cm^–2^ (20 min/cycle) show minor voltage change after 100 cycles (approx. 34 h).	[168]
PPy/FeTCPP/Co	Cycling performance at 5 mA cm^−2^ and 0.75 V charge–discharge voltage gap yields ≈ 62% round-trip efficiency, with a voltage gap increase of ≈0.1 V, after 24 h (20 min each charge and discharge session)	[170]
CoFe@N-CNWF	After 200 charge/discharge cycles at 10 mA cm^−2^, 20 min per cycle, the voltaic efficiency drops to 52.5% from 62.2%.	[174]
CPP-900	CPP-900 has outstanding endurance, enduring over 1000 cycles at 10 mA cm^−2^ in a recurrent discharge–charge cycle system.	[54]
Co_2_P/H-NPC	The initial ΔV at 2 mA cm^−2^ is 0.78 V. After 300 h (600 cycles), ΔV barely rises by 100 mV to 0.88 V.	[182]
Fe/Fe_3_C@Fe-N_x_-C	Excellent stability over 200 cycles at 5 mA cm^−2^ with a narrow discharge/charge voltage gap of ~0.87 V	[183]
(NiFe-LDH)_1_/ GA_0.18_	No decay for over 340 h. For the long-period cycling (2 h per cycle), ZABs for over 100 h, with stable charge/discharge voltage up to 53 cycles.	[85]
Fe-NBrGO	Discharge–charge cycling test at a current density of 10 mA cm^−2^—284 h cycling test, 5% reduction in performance, stable voltaic efficiency (~35%) (neutral ZABs)	[87]
Ni-MnO/r-GO	Discharge–charge cycling test at a current density of 10 mA cm^−2^—20 min each cycle—100 cycles—small overpotential increase. Round-trip overpotentials reduced voltaic efficiency by 9.1% from 0.73 V to 0.98 V at the 100th cycle.	[185]
FeCo/N-DNC	The battery has a longer cycle life (100 charge/discharge cycles) compared to the combined Pt/C + RuO_2_ battery (30 cycles).	[102]
Fe-Ni ANC@NSCA	Discharge current density of 5 mA cm^−2^ for 500 h—negligible voltage drop on both charge/discharge segments	[100]
ISG Fe-N-C	After 360 cycles for 120 h, the overpotential decreases to 0.77 V with an efficiency of 60.9%; after 660 cycles for 220 h, the overpotential increases to 0.93 V with an efficiency of 56.1%.	[121]
P-CoFe-H_3_	Initial voltaic efficiency of 57.89%; at 104th cycle, 56.54%.	[139]
CoNC-MOG-9	110 h at current density of 10 mA cm^–2^ (10 min each cycle)—negligible voltage loss.	[198]

## Data Availability

No new data were created or analyzed in this study. Data sharing is not applicable to this article.
